# The role of control in precipitating and motivating self-harm in young people: A systematic review and meta-synthesis of qualitative data

**DOI:** 10.1371/journal.pone.0325683

**Published:** 2025-06-13

**Authors:** Demee Rheinberger, Aimy Slade, Biya Tang, Ashley Hoye, Hiroko Fujimoto, Wu-Yi Zheng, Glenn Holmes, Katherine Boydell, Alison L. Calear, Helen Christensen, Samantha Tang

**Affiliations:** 1 Black Dog Institute, UNSW Sydney, Sydney, New South Wales, Australia; 2 Centre for Mental Health Research, Research School of Population Health, Australian National University, Canberra, Australian Capital Territory, Australia; 3 Faculty of Medicine and Health, UNSW Sydney, Sydney, New South Wales, Australia; King George's Medical University, INDIA

## Abstract

The last decade has seen a steady rise in self-harm rates in young people in developed countries. Understanding the experience of self-harm in young people can provide insight into what may be driving this increase. The aim of the current review was therefore to synthesise qualitative research examining precipitating and motivating factors underlying self-harm in young people. PsychInfo, Embase and Medline were systematically searched for articles published up to September 2024. A total of 50 qualitative studies (study N ranging from 3–115) were identified, and key findings were synthesised using Attride-Stirling’s Thematic Network Analysis process. The quality of included studies was assessed using a modified version of two Joanna-Briggs Institute Checklists. Control was identified as a global theme. Precipitants were informed by young people’s perception of an absence of control and the desire to gain control was identified as an underlying motivation for self-harm. The findings from this review highlight the need to support and educate young people to improve their distress tolerance, particularly in respect to situations outside of their control. Furthermore, therapeutic interventions, and training and educational programs targeting young people who self-harm and their family members might be effective interventions for self-harm in young people.

## Introduction

In Australia, rates of self-harm, defined as intentional self-injury irrespective of intention [1], have risen within the last decade. This rise has been particularly pronounced in young people aged 10–25 years, with young women accounting for the sharpest increases [2]. Similar increases in adolescent hospitalisations due to self-harm have been observed in other high-income countries around the world [3–6]. Since hospitalisations only account for a small proportion of self-harm incidents [7], it is likely that the vast majority of young people who self-harm are not receiving appropriate mental healthcare [8].

Self-harm can have profound long-term impacts, including increased likelihood of suicidal behaviour and death by suicide [9]. Individuals who engage in self-harm as adolescents are also significantly more likely to experience mental health concerns [10–12], emotion regulation difficulties [11], financial difficulties [10], and poorer life satisfaction [12] during adulthood. Given the potential life-long impacts of adolescent self-harm, it is important to understand drivers of self-harm in young people, including self-harm precipitants (i.e., the immediate circumstances or events leading to self-harm) and motivations (i.e., functions of self-harm). Doing so will aid in the development of appropriate interventions to prevent and reduce self-harming behaviour.

Qualitative research offers an opportunity to gain a deeper understanding of young peoples’ experiences of engaging in self-harm, by providing more detailed and nuanced descriptions of participant experiences. Two reviews of qualitative studies examining young peoples’ experiences of self-harm have been conducted over the past decade [13,14]. Both reviews found that emotion regulation was the most frequently reported motivator of self-harm. That is, self-harm primarily serves to release or provide control over unwanted emotions, or to elicit feeling when a young person is experiencing numbness. The reviews also found that self-harm is used as a means of inducing sensations of feeling alive, or a rush of positive feelings. Other identified functions included self-punishment, communication of distress, conflict management and group identification.

Self-harm precipitants have not been as robustly examined as self-harm functions in existing qualitative reviews. However, both Stänicke et al. [14] and Lindgren et al. [13] found that self-harm is often preceded by unwanted and difficult emotions, such as anxiety, depression, anger, shame and guilt. Self-harm has also been found to be preceded by an absence of emotion or an experience of numbness [14]. Other precipitants that have been identified by existing reviews include traumatic experiences, feelings of helplessness and interpersonal issues, such as conflicts with peers and family, a lack of support from others and loneliness [13,14].

While existing qualitative reviews provide some insights into the motivations and precipitants for self-harm, it is timely to update these reviews given recent trends in self-harm. Given significant cultural and technological shifts over this time, it is possible that motivations and precipitants of self-harm may have changed. Young people are at the forefront of new innovations and ways of living and therefore are likely to be the most impacted by these shifts. Furthermore, existing systematic reviews are limited in their methodology. While they have synthesised and summarised themes from included studies, they have not conducted secondary thematic analysis to draw out larger, overarching themes. As such, the aim of this review was to qualitatively analyse and synthesise recent qualitative literature to understand the current motivations and precipitants of self-harm in young people.

## Methods

### Search strategy and selection criteria

This systematic review adhered to Preferred Reporting Items for Systematic Reviews and Meta-Analyses (PRISMA) guidelines [15; see S1 PRISMA Checklist in S1 Checklist]. The protocol was prospectively registered with PROSPERO (registration number CRD42023429568). The volume and breadth of included studies exceeded our initial expectations, so we chose to present the results of the registered review across multiple articles. This article focuses on the results from qualitative studies identified from the review process, while the other two articles focus on quantitative studies identified in the review, including one quantitative review that examines correlates of different self-harm functions [16] and another that examines precipitants of self-harm and subgroup differences. As we did not collect new data, ethics approval was not required. However, ethics was sought by the authors of included studies.

Three electronic databases (PsycInfo, Embase & Medline) were searched using three key blocks of terms related to: i) young people aged 10–24 years, ii) self-harm and suicide, and iii) motivations and precipitants (see S2 Search strategy for the search strategy used in each database in S2 Appendix). Publication date was restricted to between 23^rd^ March 2013 and 10^th^ September 2024. No restrictions were placed on language. Reference lists of included studies and relevant past reviews were subsequently examined to identify any additional papers.

### Eligibility criteria

For the current review, eligible studies were those that examined motivations and/or precipitants for self-harm among young people and met all the following requirements:

*(i) Study design.* Eligible studies were qualitative studies (including qualitative data within mixed-methods studies), published in a peer-reviewed journal. Only first-person accounts of motivators and/or precipitants of self-harm from individuals with a history of self-harm were included (i.e., qualitative studies that only comprised of reports from parents or peers of young people with a history of self-harm were excluded). Systematic reviews, meta-analyses, case studies, conference abstracts and book chapters were excluded.
(ii) *Self-harm definition.* In line with the recent Lancet Commission on Self-Harm [1], we defined self-harm as an act with a non-fatal outcome in which an individual deliberately initiates behaviour or ingests an illicit drug or medication or non-ingestible substance or object, with the intention of causing harm to themselves.
(iii) *Population of interest.* Young people, aged 10–24 years, with a history of self-harm. Studies were included if the participant age range or mean age fell within 10–24 years. Studies that examined multiple age groups were included if they conducted analyses of interest exclusively among young people.
(iv) *Year of publication.* Our initial search included papers published between 23^rd^ March 2013 and 23^rd^ March 2023, and an updated search was conducted to include articles up to 10^th^ September 2024. The search was focused on the past 10 years given the significant increase in rates of self-harm among young people during this time period.

### Study selection

**Fig 1:**
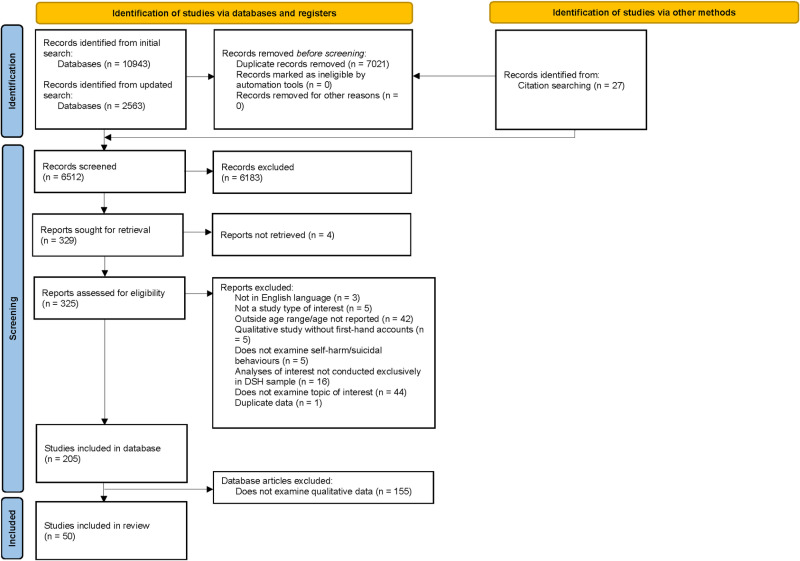
PRISMA Flow Diagram.

A PRISMA flow diagram of study identification and selection is presented in Fig 1. Following the removal of duplicates, all titles/abstracts were independently double-screened by DR, BT, ST, HF, AH, AS or WZ using Covidence [17]. Disagreements were resolved through discussion. All full-text articles were independently double-screened by ST, DR, BT, HF, AH, AS or WZ.

### Data extraction and synthesis

For each included study, data was extracted independently by two authors (DR, ST, AH, BT or GH) using Covidence. Extracted data for all included studies was checked by DR via Covidence, and disagreements were resolved through discussion. The following data was extracted for each study: bibliographic information (author, year), country, sample size, sample description (e.g., university students, in-patient sample), participant characteristics (age, gender), self-harm method examined (specific or mixed), proportion of the sample with suicidal intent, and key findings.

**Fig 2:**
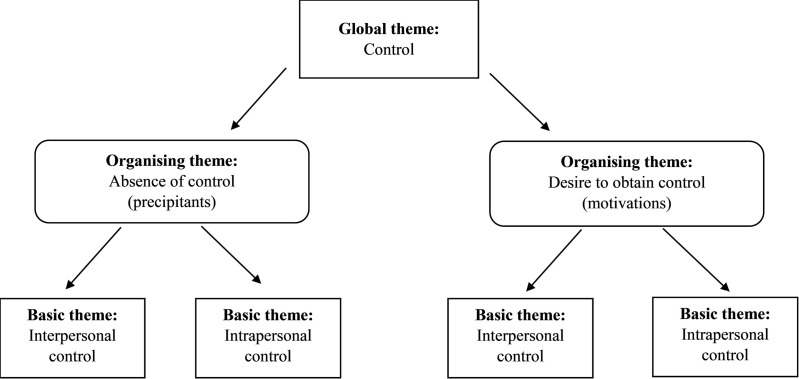
Thematic Network.

Data extracted from the included studies (N = 50) was analysed inductively following Attride-Stirling’s thematic network analysis process [18]. An inductive analysis approach was utilised to uncover patterns of shared meaning which may not be explicitly outlined within the data. Attride-Stirling’s network thematic analysis is a qualitative method that organises themes into three levels – basic themes, organising themes, and global themes – represented as web-like networks to systematically analyse and interpret textual data. First the lead author (DR) developed codes both deductively (based on a broad understanding of the area of investigation) and inductively (derived from the extracted data). Codes were then separated into either motivations or precipitants based on how this data was reported in each study and/or as inferred by the researchers during data extraction. The delineation of the data as either precipitant or motivation was to directly address the study aim. Through review and refinement of the codes, a global theme of ‘control’ was derived. The development of the global theme was interpretive and based on the data corpus, and supported by the organising themes with precipitants being experiences where young people were without control, and motivations being experiences where young people desired to obtain control. A thematic network was then created, and codes were thematically grouped into four basic themes (two each under organising themes for motivations and precipitants), with the global theme of control (see Fig 2). A narrative synthesis of the findings was used to report these findings. The analysis was led by DR and explored and refined in collaboration with ST and HC.

### Quality assessment

The quality of included studies was assessed using a modified version of the Joanna Briggs Institute (JBI) Checklists for Analytical Cross-Sectional Studies and Qualitative Studies [19; see S3 Quality assessment tool in S3 Appendix]. Five items were selected and modified based on their relevance for the current review: (1) Were the criteria for inclusion in the sample clearly defined? (2) Were the study subjects and the setting described in detail? (3) Did the qualitative methodology align with the research aims/study purpose? (4) Was the data analysis sufficiently rigorous? (5) Do the conclusions drawn align with the analysis and interpretation of the data? Studies were rated as ‘adequate’, ‘partial’, ‘poor/unclear’ for each item. Quality assessment was performed independently by two authors (DR, ST, AH, BT, or GH). Quality assessment ratings for all studies were checked by DR, and disagreements were resolved through discussion.

## Results

### Characteristics of included studies

The database and citation searches resulted in the identification of 13533 articles, 6512 after removal of duplicates. Screening of abstracts and full-text articles resulted in 50 studies being included in this review (See Fig 1). S5 and S6 Files outline reasons for exclusion of articles at the full-text screening stage and final database stage, respectively. Characteristics of included studies are reported in Table 1. The majority of studies were qualitative studies (n = 46, 92%), with the remainder being mixed methods (n = 4, 8%). In total, 986 participants were included by studies within this review, with most of the participants being female (n = 570, 57.6%). Sample sizes ranged from three to 115. Included studies were published between 2014 and 2024. Most studies were conducted in the United States of America (n = 11, 22%). Forty (80%) studies reported both motivations and precipitants of self-harm, five studies (10%) reported motivations only, and another five (10%) reported precipitants only.

**Table 1:** Characteristics of all included studies.

Author (year); country	Recruitment	Sample size (% female)	Age range in years (M, SD)	Data source	SH/NSSI/S	Precipitants	Motivations
Abeyasekera and Marecek [20]; Sri Lanka	Inpatient sample, admitted after suicide attempt	24 (100%)	15-19 (M and SD, NR)	Semi-structured interviews	Suicide attempt: self-poisoning	Interpersonal • family pressures Intrapersonal • intense negative feelings	Interpersonal • communicate with others • rebellion & revenge
Aggarwal, Patton [21]; India	Inpatient sample, admitted after self-harm	15 (67%)	15-24 (M = 19.8, SD NR)	Interviews	Self-harm	Interpersonal • family pressures • breakdown of relationships • peer self-harm • work related pressures Intrapersonal • intense negative feelings • feelings of loneliness	Interpersonal • communicate with others • rebellion & revenge
Agüero, Medina [22]; Argentina	Outpatients who self-reported self-harm or had self-harm identified during physical examination in hospital	36 (91.7%)	12-20 (M = 15.2, SD = 1.9)	Semi-structured interviews	Self-harm	Interpersonal • family pressures • abuse • breakdown of relationships • changes in home or school Intrapersonal • intense negative feelings • negative self-perception • feelings of loneliness	Interpersonal • communicate with others Intrapersonal • regulate unwanted emotions • death or escape • induce positive emotions • self-punishment
Almeida, Barber [23]; USA	Inpatient sample, admitted after suicide attempt	20 (75%)	13-17 (M = 14.4, SD NR)	Semi-structured interviews	Suicide attempt	NA	Intrapersonal • death or escape^*^
Balaji, Mandhare [24]; India	Inpatient sample, admitted after suicide attempt	47 (57%)	15-29 (M = 22, SD NR)	Semi-structured interviews	Suicide attempt	Interpersonal • family pressures • breakdown of relationships • abuse • work related pressures Intrapersonal • intense negative feelings • negative self-perception • feelings of loneliness • feelings of powerlessness • illness or injury	Interpersonal • communicate with others Intrapersonal • death or escape • self-punishment
Chen, Wang [25]; China	Outpatient and inpatient sample with 2 or more episodes of self-harm	22 (86.4%)	12-24 (M = 18.0, SD = 3.0)	Semi-structured interviews	Self-harm	Interpersonal • family pressures • breakdown of relationships • bullying • peer self-harm Intrapersonal • intense negative feelings • negative self-perception	Intrapersonal • regulate unwanted emotions • induce positive emotions
Cronemberger and Silva [26]; Brazil	Outpatient sample with experience of NSSI	5 (100%)	14-24 (M = 17, SD = 4.0)	Semi-structured interviews	NSSI	Interpersonal • family pressures • abuse • bullying • peer self-harm Intrapersonal • intense negative feelings • negative self-perception • feelings of loneliness	Interpersonal • communicate with others Intrapersonal • regulate unwanted emotions • induce positive emotions
Curtis [27]; New Zealand	Community sample recruited online and through flyers at university and service providers with a self-identified history of NSSI	19 (100%)	16-25 (M and SD NR)	Unstructured interviews	NSSI	Interpersonal • abuse • bullying • peer self-harm Intrapersonal • Intense negative feelings	Interpersonal • communicate with others Intrapersonal • regulate unwanted options • death or escape^*^
Čuš, Edbrooke-Childs [28]; Austria	Outpatient sample of recent, repetitive instances of NSSI	15 (100%)	12-18 (M = 15.2, SD = 1.6)	Semi-structured interviews	NSSI	Intrapersonal • intense negative feelings • feelings of powerlessness	Intrapersonal • regulate unwanted options • self-punishment
Doyle, Sheridan [29]; Ireland	School based sample	103 (72.8%)	15-17 (M and SD NR)	Open-ended survey questions	Self-harm	Interpersonal • family pressures • breakdown of relationships Intrapersonal • negative self-perception	Interpersonal • communicate with others Intrapersonal • regulate unwanted emotions • death or escape
Grandclerc, Spiers [30]; France	Inpatient sample admitted after suicide attempt or NSSI	18 (100%)	12-21 (M = 16.5, SD NR)	Semi-structured interviews over two time points	Suicide attempt or NSSI	Intrapersonal • intense negative feelings	Interpersonal • communicate with others • rebellion & revenge Intrapersonal • regulate unwanted emotions • death or escape • induce positive emotions • self-punishment
Guest, Copello [31]; United Kingdom	Outpatient sample with a history of suicide attempt	7 (85.7%)	16-24 (M = 20, SD NR)	Semi-structed interviews	Suicide	Interpersonal • family pressures • breakdown of relationships • abuse • bullying • work related pressures Intrapersonal • intense negative feelings • feelings of loneliness	Interpersonal • communicate with others Intrapersonal • death
Gulbas, Hausmann-Stabile [32]; USA	Inpatient and outpatient sample with a history of suicide attempt and/or NSSI	Overall sample: 83 (100%) NSSI only: 18 (100%) Suicide attempt only: 29 (100%) NSSI & SA: 8 (100%)	Range NR (M = 15.3, SD = 1.9) NSSI only: (M = 15, SD = 1.4) Suicide attempt only: (M = 15.5, SD = 2.0) NSSI & suicide attempt: (M = 15, SD = 2.3)	Interviews	NSSI and Suicide attempt	Interpersonal • family pressures • breakdown of relationships • abuse • bullying • changes in home or school Intrapersonal • intense negative feelings • negative self-perception • feelings of powerlessness	Interpersonal • communicate with others • rebellion & revenge Intrapersonal • regulate unwanted emotions • death or escape
Gulbas and Zayas [33]; USA	Inpatient and outpatient sample with a self-identified history of suicide attempt	10 (100%)	Range NR (M = 15.7, SD NR)	Open-ended interviews	Suicide attempt	Interpersonal • family pressures • breakdown of relationships • abuse Intrapersonal • negative self-perception • feelings of loneliness	Intrapersonal • death or escape
Hahm, Gonyea [34]; USA	Community sample with a self-identified history of self-harm or suicide attempt	16 (100%)	18-30 (M = 22, SD NR)	Semi-structed interviews	Self-harm and/or suicide attempt	Interpersonal • family pressures	Interpersonal • communicate with others • rebellion & revenge Intrapersonal • regulate unwanted emotions • self-punishment
Hetrick, Subasinghe [35]; Australia	Outpatient sample with a history of self-harm or suicide attempt. Chain referral sampling – participants invited others they felt might be eligible	7 (71.4%)	18-24 (M = 20.6, SD = 2.2)	Semi-structured interviews	Self-harm	Interpersonal • family pressures • breakdown of relationships • bullying • peer self-harm • work related pressures Intrapersonal • intense negative feelings • negative self-perception • feelings of loneliness	NA
Hird, Boyes [36]; Australia	University sample of trans people with a history of NSSI	20 (5%)	14-25 (M = 18.89, SD NR)	Semi-structured interviews	NSSI	Interpersonal • family pressures • bullying Intrapersonal • intense negative feelings • negative self-perception • feelings of powerlessness	Interpersonal • communicate with others Intrapersonal • regulate unwanted emotions • self-punishment
Holliday and Vandermause [37]; USA	Inpatient sample admitted after suicide attempt	6 (83.3%)	15-19 (M and SD NR)	Interviews	Suicide attempt	NA	Interpersonal • communicate with others Intrapersonal • regulate unwanted emotions • death or escape
Holliday, Brennan [38]; England	Outpatient presenting with self-harm	22 (63%)	11-17 (M and SD NR)	Transcribed video recordings of family-therapy sessions	Self-harm	Interpersonal • family pressures Intrapersonal • negative self-perception • feelings of loneliness	Interpersonal • communicate with others Intrapersonal • regulate unwanted emotions • death or escape • induce positive emotions
Latakienė and Skruibis [39]; Lithuania	Inpatient sample admitted for suicide attempt	3 (100%)	13-17 (M = 15, SD = 2.0)	Semi-structured interviews	Suicide attempt	Interpersonal • family pressures • breakdown of relationships Intrapersonal • intense negative feelings • feelings of loneliness	NA
Lockwood, Townsend [40]; England	University sample with a history of self-harm behaviour	15 (93.3%)	16-22 (M = 17.4, SD NR)	Semi-structured interviews and card-sorting tasks	Self-harm	Intrapersonal • intense negative feelings	Intrapersonal • regulate unwanted emotions • death or escape
Marzetti, McDaid [41]; Scotland	Community sample	24 (45.8%)	16-24 (M = 19.6, SD NR)	Semi-structured interviews	Self-harm or suicide attempt	NA	Intrapersonal • regulate unwanted emotions • death or escape
McAndrew and Warne [42]; United Kingdom	Community sample with a history of self-harm	7 (100%)	13-17 (M and SD NR)	Narrative interviews	Self-harm	Interpersonal • family pressures • breakdown of relationships • bullying • peer self-harm Intrapersonal • intense negative feelings • negative self-perception	Intrapersonal • regulate unwanted emotions
McClelland, Evans [43]; Scotland	University sample with a history of suicide attempt	10 (50%)	20-25 (M = 22.5, SD NR)	Semi-structured interviews	Suicide attempt	Interpersonal • family pressures • breakdown of relationships • changes in home or school Intrapersonal • negative self-perception • feelings of loneliness	NA
Miller, Redley [44]; England	Outpatient sample with a history of self-harm	9 (100%)	13-17 (M and SD NR)	Semi-structured interviews	Self-harm	Interpersonal • family pressures • breakdown of relationships • abuse • bullying Intrapersonal • negative self-perception	Interpersonal • communicate with others Intrapersonal • regulate unwanted options • death or escape^*^ • induce positive emotions
Moraes, Moreira [45]; Brazil	Outpatient sample with a history of self-harm	7 (100%)	13-18 (M = 15, SD NR)	Focus groups	Self-harm	Interpersonal • family pressures • breakdown of relationships • abuse • bullying • peer self-harm	Intrapersonal • regulate unwanted emotions • death or escape
Mughal, Chew‐Graham [46]; England	Community sample	13 (92.3%)	19-25 (M = 22, SD NR)	Semi-structured interviews	Self-harm	NA	Interpersonal • communicate with others Intrapersonal • regulate unwanted emotions • death or escape^*^ • induce positive emotions • self-punishment
Naz, Naureen [47]; Pakistan	Inpatient sample admitted for self-harm	16 (56.3%)	14-17 (M and SD NR)	Semi-structured interviews	Self-harm	Interpersonal • family pressures Intrapersonal • intense negative feelings • feelings of loneliness	Interpersonal • communicate with others Intrapersonal • death or escape
O’Brien, Nicolopoulos [48]; USA	Inpatient sample admitted for suicide attempt	20 (75%)	13-17 (M = 14.4, SD NR)	Semi-structured interviews	Suicide attempt	Interpersonal • family pressures • breakdown of relationships • abuse Intrapersonal • intense negative feelings • negative self-perception • feelings of loneliness • feelings of powerlessness	Intrapersonal • regulate unwanted emotions • death or escape
Orri, Paduanello [49]; Italy	Inpatient and outpatient sample with a history or self-harm while an adolescent or young adult	16 (50%)	17-25 (M = 19.8, SD = 2.1)	Semi-structured interviews	Self-harm	Interpersonal • family pressures • breakdown of relationships Intrapersonal • intense negative feelings • negative self-perception • feelings of loneliness • feelings of powerlessness	Interpersonal • communicate with others • rebellion & revenge Intrapersonal • death or escape
Quarshie, Waterman [50]; Ghana	School and homeless sample	36 (72.2%)	In school: 15–20 (M = 17, SD NR) Homeless: 13-19 (M = 16, SD NR)	Semi-structured interviews	Self-harm	Interpersonal • family pressures • changes in home or school Intrapersonal • feelings of powerlessness	Interpersonal • communicate with others • rebellion & revenge Intrapersonal • regulate unwanted emotions
Santo and Dell’Aglio [51]; Brazil	Outpatients with a history of self-harm	4 (NR)	13-17 (M = 14.5, SD = 1.9)	Semi-structured interviews	Self-harm	Interpersonal • family pressures • breakdown of relationships • bullying • peer self-harm Intrapersonal • intense negative feelings • negative self-perception • feelings of loneliness	Intrapersonal • regulate unwanted emotions • death or escape • self-punishment
Shahwan, Zhang [52]; Singapore	Outpatients with self-reported history of NSSI	20 (70%)	17-19 (M and SD NR)	Semi-structured interviews	NSSI	Interpersonal • work related pressures	Interpersonal • communicate with others Intrapersonal • regulate unwanted emotions • induce positive emotions • self-punishment
Simões, Oliveira [53]; Brazil	Outpatient sample with a lifetime history of suicide attempt	10 (90%)	12-17 (M and SD NR)	Semi-structured interviews	Suicide attempt	Interpersonal • family pressures • breakdown of relationships • abuse • peer self-harm • changes in home or school Intrapersonal • negative self-perception	Intrapersonal • regulate unwanted emotions • death or escape
Sloan, Moulding [54]; Australia	Outpatient sample currently engaging in self-harm, substance abuse and/or binge/purge behaviours	Overall sample: 12 (66%) SH sample: 5 (NR)	Overall sample: 16–25 (M = 19, SD = 2.95) SH sample NR	Semi-structured interviews	NSSI	Interpersonal • family pressures • breakdown of relationships Intrapersonal • intense negative feelings	Intrapersonal • regulate unwanted emotions
Stänicke [55]; Norway	Outpatient sample with a history of self-harm	19 (100%)	13-18 (M = 15.9, SD NR)	Semi-structured interviews	Self-harm	Interpersonal • family pressures • breakdown of relationships • abuse • bullying Intrapersonal • intense negative feelings • negative self-perception • feelings of loneliness	Interpersonal • communicate with others Intrapersonal • regulate unwanted emotions • induce positive emotions • self-punishment
Stänicke, Haavind [56]; Norway	Outpatient sample with documented history of self-harm	19 (100%)	13-18 (M = 15.9, SD NR)	Interviews	Self-harm	Interpersonal • family pressures • breakdown of relationships • bullying • peer self-harm Intrapersonal • intense negative feelings • negative self-perception	Intrapersonal • regulate unwanted emotions
Stradomska, Wolińska [57]; Poland	Inpatient sample admitted for suicide attempt	115 (NR)	13-19 (M and SD NR)	Structured interview	Suicide attempt	Intrapersonal • intense negative feelings • feelings of powerlessness • illness or injury	Intrapersonal • death or escape
Sukhawaha, Arunpongpaisal [58]; Thailand	Inpatient sample admitted for suicide attempt	12 (75%)	15-18 (M and SD NR)	Interviews	Suicide attempt	Interpersonal • family pressures • breakdown of relationships Intrapersonal • intense negative feelings	NA
Szlyk, Gulbas [59]; USA	Inpatient and outpatient sample with a history of suicide attempt	10 (100%)	Range NR (M = 15.4, SD NR)	Interviews	Suicide attempt	Interpersonal • family pressures • abuse	NA
Taliaferro, Almeida [60]; USA	Inpatient sample with a history of suicide attempt and NSSI	15 (86.7%)	13-17 (M = 14.4, SD NR)	Structured interviews	Suicide attempt & NSSI	NA	Intrapersonal • regulate unwanted emotions • death or escape • induce positive emotions • self-punishment
Tan, Rehfuss [61]; Singapore	Outpatient sample with a history of NSSI in prior year	NSSI sample: 30 (60%) Qualitative NSSI sample: 6 (NR)	NSSI sample: 13–19 (M = 16.3, SD = 1.7) Qual sample NR	Semi-structured interviews	NSSI	Interpersonal • family pressures Intrapersonal • intense negative feelings • negative self-perception	Intrapersonal • regulate unwanted emotions
Tan, Tam [62]; Maylasia	University students who had participated in prior study and endorsed self-injury in prior 12 months	7 (85.7%)	18-22 (M = 20.3, SD NR)	Semi-structured interviews	Self-harm	Interpersonal • family pressures • breakdown of relationships • abuse Intrapersonal • intense negative feelings • negative self-perception • feeling of powerlessness	Intrapersonal • regulate unwanted emotions • death or escape • induce positive emotions • self-punishment
Tillman, Prazak [63]; USA	Community sample of 8^th^ graders with a history of NSSI and who had received therapeutic intervention	6 (100%)	Range NR (M = 13.8, SD = 0.41)	Interview	NSSI	Interpersonal • breakdown of relationships • abuse • Peer self-harm Intrapersonal • intense negative feelings • negative self-perception	Interpersonal • communicate with others Intrapersonal • regulate unwanted emotions • induce positive emotions
Tingey, Cwik [64]; USA	Community sample with a history of suicide attempt	22 (NR)	13-19 (M and SD NR)	Semi-structured interviews	Suicide attempt	Interpersonal • family pressures • breakdown of relationships • bullying • peer self-harm Intrapersonal • intense negative feelings • negative self-perception • feelings of powerlessness	Interpersonal • communicate with others
Wadman, Clarke [65]; United Kingdom	Community sample with a history of repeated self-harm	6 (83.3%)	19-21 (M = 20, SD NR)	Semi-structured interviews	Self-harm	Interpersonal • family pressures	Intrapersonal • regulate unwanted emotions • induce positive emotions • self-punishment
Wadman, Vostanis [66]; United Kingdom	Community sample who had self-harmed in prior 6 months	14 (100%)	13-18 (M = 16, SD NR)	Semi-structured interviews	Self-harm	Interpersonal • family pressures • abuse • bullying	Intrapersonal • regulate unwanted emotions
Williams, Arcelus [67]; UK, USA and Isreal	Community sample of LGBTQI youth with a history of SH	19 (52.6%)	16-25 (M = 21.2, SD = 2.7)	Semi-structured interviews	Self-harm	Interpersonal • family pressures • breakdown of relationships • abuse • bullying Intrapersonal • negative self-perception • illness or injury	Interpersonal • communicate with others Intrapersonal • death or escape • self-punishment
Wong and Chung [68]; Hong Kong	University mental health service users with a history of engaging in NSSI	7 (100%)	18-21 (M = 19.9, SD = 1.4)	Semi-structured interviews	NSSI	Interpersonal • breakdown of relationships Intrapersonal • negative self-perception • feelings of loneliness	Interpersonal • communicate with others Intrapersonal • regulate unwanted emotions • induce positive emotions
Zhu, Miao [69]; China	Outpatient sample with a history of NSSI	18 (61.1%)	12-18 (M = 13.89, SD = 1.7)	Semi-structured interviews	NSSI	Interpersonal • family pressures • abuse • bullying • peer self-harm Intrapersonal • intense negative feelings • negative self-perception • feelings of loneliness • feelings of powerlessness • illness or injury	Interpersonal • communicate with others • rebellion & revenge Intrapersonal • regulate unwanted emotions • death or escape • induce positive emotions • self-punishment

**Note:** SH = self-harm, NSSI = non-suicidal self-injury, S = suicide, NR = not reported.

* protection against suicide only.

### Qualitative results

Qualitative analysis was conducted on the findings of the 50 included articles. An overarching theme of “control” was identified with subthemes of ‘absence of control’ as the overarching circumstance (i.e., precipitant) that triggers self-harm, and ‘desire to obtain control’ as the high-level influence on the motivation to engage in self-harm. Both these subthemes were further segmented into interpersonal (socially derived) and intrapersonal (self-derived) elements, see Table 2.

**Table 2:** Theme structure.

Global theme: control N = 50		
**Organising theme:**	**Basic themes:**	**Code**
Absence of control (precipitant) N = 45	Interpersonal control N = 41	Family pressures n = 37
		Breakdown of relationships n = 27
		Abuse n = 18
		Bullying n = 17
		Peer self-harm n = 13
		Changes in home or school n = 5
		Work related pressures n = 5
	Intrapersonal control N = 39	Intense negative feelings n = 30
		Negative self-perception n = 26
		Feelings of loneliness n = 18
		Feelings of powerlessness n = 11
		Illness or injury n = 4
Desire to obtain control (motivation) N = 45	Interpersonal control N = 26	Communicate with others n = 26
		Rebellion & revenge n = 8
	Intrapersonal control N = 42	Regulate unwanted emotions n = 34
		Death or escape n = 26^*^
		Induce positive emotions n = 15
		Self-punishment n = 15

NOTE: ^*^Eight studies report that self-harm was protective against suicide attempt.

#### Absence of control (precipitant).

The events (i.e., precipitants) leading up to self-harm in young people appeared to involve a perceived absence of control, including an absence of interpersonal and interpersonal control.

*Basic theme 1.1 – Absence of interpersonal control.* This subtheme explores the precipitants which are due to an absence of control brought about due to interactions or experiences with others. A total of 41 studies (82%) are included in this section. Interpersonal precipitants identified include family pressure, breakdown of key relationships, abuse, bullying, peer self-harm, changes in the home or school and work-related pressures.

*Family pressures –* Family pressures was the most common precipitant, having been reported across 37 studies. Such pressures culminated in the young person feeling as though they are unable to control or leave dysfunctional or harmful family situations. Studies discussed difficult relationships with parents and other family members [20,22,24–26,29,31–36,38,42–45,50,51,53,56,59,66,67,69], including conflict with parents or other family members [20–22,24,25,32–36,39,44,45,47,48,51,53,54,58,64–66]. Conflict arose in relation to a variety of issues, such as the young person’s mental health or self-harm behaviours [29,33–35,58,66], a lack of affection or care from parents [22,24,32,33,39,43,45,50,53,55,56,61], dysfunctional parenting [20,49,50,64,66,69], and a lack of trust or acceptance from family members [20,24,26,33]; *“She [mother] doesn’t accept me for who I am. And that she’s very negative toward me.”* [32, pg. 309]. Many studies also identified failing to meet parents’ expectations for academic achievement or career progress [21,33,35,59,61,62,69], nonadherence to traditional gender expectations [20,50,59], or disclosure of LGBTQ+ status [36,67] as a factor leading to self-harm. One study found that criticisms from parents was particularly prevalent in young people who attempted suicide [32].

Studies also described young people not feeling supported by family members [22,24,35,36,45,49,51,59], poor communication between young people and their parents [33,35,44,48,56,59,66], and young people being invalidated by family members [21,24,51,59,61,69] as precipitants leading up to self-harm.

Difficult family events such as divorce, witnessing domestic violence, parental health issues, and alcohol and drug use [20,24–26,31–33,39,45,51,55,56,59,64,69], being part of the foster care system [39], and caring for family members who are disabled or ill [24,67] were also identified as precipitating circumstances for self-harm, and reflect situations in which young people experience a loss of control around their home and family context.

*Breakdown of relationships.* A total of 27 studies identified the breakdown or ending of key personal relationships including romantic relationships [21,24,25,31,35,39,43,53,54,58,68], close friendships [21,53], and key familial relationships [29,32,39,51,62], as a precipitant to self-harm. In terms of friendship breakdowns, these often arose from conflict with friends [21,22,24,25,29,35,39,42,44,48,49] and the negative impact of young people’s own declining mental health [43,51] or suicide attempts [64]. The breakdown of relationships, including family and friends, as a result of death was also identified as a precipitant for self-harm [22,24,32,33,42,45,51,53,55,58,63,64]. The breakdown or loss of key relationships was often outside of the young person’s control, or perceived to be, and had profound unwanted impacts on the young person, leading them to self-harm.

*Abuse.* Abuse, be it psychological, physical, verbal, or sexual in nature, was identified in 18 studies as a factor precipitating self-harm [22,24,26,27,31–33,44,45,48,53,55,59,62,63,66,67,69]. In such circumstances, control is held by perpetrators of abuse. In one study, young people reported that the pressure to keep abuse hidden was a precipitant to self-harm [59].

*Bullying.* The inescapable and uncontrollable experience of being bullied was identified as a precipitant in 17 studies [25–27,31,32,35,36,42,44,45,51,55,56,62,64,66,67,69]. Wadman et al. [66] reported bullying was a long-term, background stressor which contributed to the self-harm when experienced alongside other stressors. Some studies noted that bullying occurred online as well as in person [25,35], highlighting the young peoples’ inability to escape from the bullying. Hird et al. [36] reported exposure to, and fear of future instances, of transphobia directed toward trans young people precipitated self-harm. Young people also reported using self-harm as a reason to leave a situation where they expected to be targeted by others [36].

*Peer self-harm.* Exposure to peers who self-harmed was sometimes reported to precipitate self-harm, with some young people being encouraged to self-harm by peers who had found it helpful to gain control over their issues; *“I asked my desk mate how to get out of a low mood. He said he was depressed as well and suggested cutting hands with a compass as a solution. I was curious so I tried it.”* [25, pg. 3]. Exposure to self-harm took the form of hearing about others’ experiences, seeing scars on others or images of self-harm, or reading stories about how self-harm was helpful [21,27,35,42,45,51,53,56,63,64,69]. Young people were also exposed to self-harm via online social media platforms [25,26,35,45,56], with discussions or images of self-harm methods particularly triggering [35]. Sometimes, exposure to peer self-harm created a sense of competition in which young people needed to increase the intensity of their own self-harm [35].

*Changes in home or school.* Changes in the home or school environments were the most obvious and physical representation of the absence of control within the articles identified in this review. Studies found that changing place of residence or school was a time of great upheaval [22,53] and led to feelings of fear and insecurity [53], a loss of social supports [32,43,53], and adjustment to new living conditions and requirements [50], which increased the propensity for self-harm behaviours.

*Work related pressures.* In five studies, a lack of control in the workplace context were mentioned as self-harm precipitants. Young people engaged in deliberate self-harm in response to making a mistake at work [52], conflict with colleagues [21], workplace pressures [35], losing employment [24,31], or sustained unemployment [24].

*Basic theme 1.2 – Absence of intrapersonal control.* This subtheme outlines self-harm precipitants which are due to the young people feeling as though they are unable to control their own thoughts or feelings and includes findings from 39 studies (86.7% of studies discussing precipitants). The precipitants in this subtheme included intense negative feelings, negative perception of self, feelings of loneliness, feelings of powerlessness, and illness or injury.

*Intense negative feelings.* Experiencing intense negative emotions, such as hopelessness, guilt, sadness, shame, anger, guilt, and anxiety were reported by 60% of the studies in this review [20–22,24–28,30–32,35,36,39,40,42,47–49,51,54–58,61–64,69]. Studies reported that young people felt heightened emotional distress [47], chaotic thoughts and feelings [55], and difficulty controlling or expressing emotions and thoughts [24,51].

Numerous studies reported poor mental wellbeing precipitated self-harm behaviour including depression, anxiety, eating disorders, ongoing mental health problems, poor sleep hygiene, rumination, and suicidal crisis [22,24,25,27,30,31,35,48,56,58,69].

*Negative self-perception.* For young people, difficulties experiencing or altering negative perceptions of self were a significant intrapersonal influence on self-harm and was identified in 26 studies [22,24–26,29,32,33,35,36,38,42–44,48,49,51,53,55,56,61–64,67–69]. A sense of worthlessness was commonly reported [29,32,56,64], and young people were negatively impacted by impressions that they were inferior to others, including their peers [35,68], or a disappointment or burden to others [24,35,38,48,55,68]. Failure to meet their own goals, ‘doing something wrong’, and associated guilt were common [22,33,55,56,62] as was dissatisfaction with one’s appearance [25,53,56]. Studies also reported that young people expressed low self-esteem [22,26] and related anxiety; *“… it caused me to be even more anxious because I was thinking, if I struggled for this year, how am I going to manage the next year?”* [43, p.g. 9]. Self-harm in response to stress induced by underperformance in academic endeavours was also common [22,24–26,32,33,35,42–44,48,51,61,62,67–69]. One participant in Gulbas et al., [32] articulated this clearly: *“School was getting on my nerves. I was really stressed and I decided I would do a cut every day.”* (pg. 310). Two studies identified internalised negative perceptions for those who identified as a member of the LBGTQ+ community, body dissatisfaction, and difficulty accessing medically assisted gender transitions [36,67].

Negative thoughts of self were often worse after interpersonal conflict [48] and were exacerbated by feelings of not being accepted or cared for by others [49]. This negative self-perception was often difficult for participants to manage [55].

*Feelings of loneliness.* Perceptions of loneliness also precipitated incidents of self-harm [22,24,26,31,35,38,39,43,47,49,51,55,68,69]. Young people reported feeling uncared for by others [33,68] and misunderstood [31,35,43,49,51], and experienced periods of social disconnection or isolation [21,43]. This sense of social isolation was arrived at through strained relationships with others, typified by a lack of attention or support [39,48], or difficulties forming social connections [33]. Young people also reported feeling isolated due to nonconformity with social norms, for instance due to LGBTQI+ status [35,36,51]. Social disconnection also led to feelings of helplessness and entrapment [43].

*Feelings of powerlessness.* Feelings of powerlessness or helplessness were often cited as precipitants of self-harm [24,28,32,36,48–50,57,62,69]. This included feeling trapped in one’s own feelings or a situation [48,49], a perceived inability to cope with such feelings or challenging situations [62], unequal power dynamics in relation to gender roles [50], excessive parental control which restricted autonomy [69], and worry about the future [64].

*Illness or injury.* Self-harm sometimes occurred in the context of uncontrollable physical pain or injury. Physical pain such as headaches, fever, and abdominal pain were sometimes reported prior to a suicide attempt [24,57,69]. Such physical pain also negatively impacted mood and behaviour, altered young people’s mental state, and reduced capacity for thought [24]. Chronic pain or illness created stressors for young people, some of whom isolated themselves in response to the pain [67].

#### Desire to regain control (motivations).

Young people were motivated to engage in self-harm by a desire to regain control, both in regard to their interactions with others, and their internal states. Forty-five studies (90%) discussed motivations for self-harm.

*Basic theme 2.1 – Desire to regain interpersonal control.* Twenty-six studies reported that young people engaged in self-harm in order to communicate with or alter the perception or feelings others. This subtheme discusses the use of self-harm to communicate with others, and as an act of rebellion and revenge, and to assist with group identification.

*Communicate with others.* Twenty-one studies reported that young people engaged in self-harm as a way of communicating their distress with others [22,26,30–32,34,36–38,44,46,47,49,50,52,55,63,64,67–69]. Such communication served as a means of controlling others’ responses. Sometimes young people were hopeful that self-harm would encourage others to take their distress seriously [30,32,34,36,38,69] – *“It was a little bit the idea in my body that told me: go ahead, do something serious and then like that, you’ll get taken care of, someone will take care of you...”* [30, pg. 9]. Curtis [27] found that for individuals who were feeling suicidal, non-suicidal self-injury served as a cry for help. However, young people were left disappointed when people did not react or provide support after discovering the self-harm [34,55].

Six studies found that young people were motivated to self-harm in order to influence another person’s behaviour [20,21,24,32,50,69]. However, a mixed method study found gaining attention from others was the least commonly endorsed motivation [29]. Furthermore, Holliday et al., [38] reported that sometimes, for young people, communicating their distress via self-harm was not about seeking attention, but rather a way to express themselves.

*Rebellion & revenge.* Through acts of self-harm, young people sought to control how others responded to, or acted towards them, although only eight studies (17.8% of studies examining motivations) reported on this self-harm motivation. In these studies, young people engaged in self-harm as an act of rebellion [69], including against parental control or punishment [30,34,50], prearranged marriage [20], or to push people away [30].

Self-harm was also sometimes motivated by a desire *“to punish or enact influence over parents”* [32, pg. 306], or to make people feel guilty or remorseful for their mistakes or carelessness [49]. One study found that some young people sought revenge on their romantic partner and engaged in self-harm to prove they would kill themselves for their partner [21].

*Basic theme 2.2 – Desire to regain intrapersonal control.* This subtheme discusses the motivations to engage in self-harm behaviours with the goal of gaining control of their internal state (e.g., thoughts and feelings) and consisted of findings from 93.3% of included studies reporting motivations (n = 42). The intrapersonal motivations included regulation of unwanted emotions, death, inducing positive emotions, and self-punishment.

*Regulate unwanted emotions.* Regulation or control of unwanted, often distressing emotions was the most frequently reported motivation for engaging in self-harm (n = 34). Studies reported that young people engaged in self-harm to control and manage overwhelming, unwanted emotions, such as low mood and loneliness [22,25–30,32–34,36–38,40,42,44–46,48–56,60–63,65,68,69]. Studies indicated that self-harm provided a release of emotions [28,30,37,38,51,52,54,66,68], the ability to convert emotional distress into physical pain [26,32,38,44,46,48,51,54,55,61], and a distraction from unwanted emotions [26,29,36,38,40,44,46,51,54,55,60,68,69]. Self-harm allowed young people to clear their minds [68] and *“focus on other things”* [40, pg. 11], and/or overwrite painful memories and thoughts [51,62,68]. Studies also indicated that self-harm is used to elicit emotion in times of emotional numbness [29,38,41,44,52,60,69]: *“I subject myself to pain to experience suffering, to remind myself that I am capable of feelings, for currently, I am engulfed in numbness”* [69, pg. 6].

*Death or escape.* Many studies outlined desire for death as a motivation for self-harming behaviours [22,24,29,31,32,37,38,40,41,45,47–49,51,53,60,62,67]. Young people sought to control their experience by deciding when and how they would die. Suicide was viewed as an appropriate response to young peoples’ inner pain and distress [31,32,41,48,53]: *“I was having a very bad day, and I didn’t wanna be alive anymore. I just wanted to be dead”* [48, pg. 277], or as a means of escape [24,33,49,69]. One study found that young people experienced a sense of relief prior to suicide attempt as they believed their problems would be solved and left behind [57].

For some young people, the intention behind self-harm was not death but an intention to disappear [37,38,40]. Some participants fantasised about the self-harm leading to death, although did not intentionally attempt suicide [30,60]. Eight studies noted that self-harm was used as a way to protect the young person from suicide, suggesting that self-harm was a way of controlling suicidal thoughts and urges [23,27,38,40,41,44,46,60].

*Induce positive emotions.* Self-harm provided young people with a way of controlling their emotion, by inducing a positive emotional response [25,30,52,60,68]. The experience of seeing blood when engaging in cutting reinforced a sense of ‘being alive’ [30,68], or induced a sense of calm, relief or comfort [22,25,26,38,44,52,55,62,63,65,68,69]. These positive responses persisted regardless of the number of self-harm incidents [25,62]. Studies also found that young people experienced positive emotions from the self-care necessary to treat injuries after engaging in self-harming behaviour [46,68].

*Self-punishment.* Self-punishment – as a means of controlling how young people felt about themselves and their actions – was identified as a motivation for engaging in self-harm in approximately a third of all studies [22,28,30,34,36,46,51,52,55,60,62,65,67]. Studies found that young people self-punished via self-harm in response to self-criticism [28,30,34,46,55,62,67], or to *“justify internal pain and distress”* [46, p.1184, 65]. Self-punishment was also a response to feelings of guilt [52,55,60], punishment for prior self-harm instances [62], for not conforming to gender or sexual norms and perceived sinfulness [36,67]; *“When I was self-harming it was often like a self-flagellation almost, like I’m doing this to absolve myself. It was mainly that it felt sinful to do what I was doing.”* [36, pg. 4], or because of conflict with parents [52,65]. Some young people engaged in self-harm to control anger that they were feeling for another person by turning it inwards, rather than outwardly expressing it [24,52,69].

### Quality assessment

The quality of included studies is outlined in S4 Appendix. Most studies adequately described their methodology (86%). Approximately three quarters of the studies adequately reported conclusions which were in alignment with the study design and results. The inclusion criteria were described adequately in 70% of included studies. Less than half of the studies (42%) adequately described the study setting or adequately reported engaging in rigorous analysis procedures.

## Discussion

The current review explored the motivations and precipitants of self-harm in young people, building upon existing literature by thematically analysing the corpus of recent qualitative literature. It is the first review in this area which incorporates a meta-synthesis approach utilising Thematic Network Analysis [18], which allowed for the identification of control as an overarching factor in young people’s self-harm. More specifically, we found that young people engage in self-harm as a way of obtaining control when they feel as though they are out of control. The review extends upon existing literature by establishing control as not only a motivation or function of self-harm, but also a precipitant of self-harm, with young people engaging in self-harm in response to instances they feel unequipped to manage. It is likely that control has always influenced self-harm for young people, given the motivations and precipitants reported in this review do not differ significantly from those reported in previous reviews [13,14]. It remains unclear why self-harm rates are increasing in young people, with included studies not finding any evidence to indicate that significant cultural or technological shifts are influencing self-harm or the experience of ‘control’ in young people.

Family pressures such as conflict, high expectations and a lack of support were frequently identified as precipitants to self-harm, which is consistent with existing literature [13,14]. Adolescence is a fundamental period for identity formation [70]. Our findings suggest that young people are attempting to understand their role in interpersonal situations, particularly within the family, and can struggle with how little control they have in such situations, leading to self-harm. Since family pressures have been identified as the most frequently reported precipitant to a first instance of self-harm [71], it is important to understand and address the family dynamics and parent-child relationships that lead to self-harm. Improved parental education around mental health and why adolescents turn to self-harm could improve the parent/child relationship, may reduce the impact of family pressures on young people and reduce the perceived necessity of self-harm to manage these situations [72]. Furthermore, family pressures are likely influenced by other factors such as parental mental health problems, financial instability, and drug or alcohol dependence [73]. Interventions and policies that address such issues, such as parenting programs (e.g., Triple P, [74]) and access to affordable mental health support, may therefore play an important role in preventing and reducing self-harm among young people.

The presence of unpleasant and unwanted emotions was frequently identified as a precipitant to self-harm, again in alignment with previous reviews [13,14]. This is not surprising as studies have demonstrated a strong link between self-harm behaviours and mental health difficulties [75,76], as well as emotions such as stress [77], aggression [78] and fear [79]. Given that a significant number of young people report experiencing these unwanted emotions, but only a proportion engage in self-harm behaviours [80], a more nuanced examination may assist in the development of more appropriately targeted interventions to address the experience of unwanted emotions in young people at risk of self-harm. For example, future qualitative studies could explore how the experience of these emotions may differ between young people who do and do not engage in self-harm; it is possible that those who do engage in self-harm experience such emotions as less controllable than those who do not engage in self-harm. Additionally, future longitudinal studies may be required to establish how the experience of unwanted emotions and loss of control more generally has shifted over time; such research may allow for greater insight into what is driving the upward trend in self-harm among young people.

Following from this, the desire to control and regulate unwanted emotions was the most frequently reported motivation for self-harm in this review. Included studies reported that young people felt that self-harm could alleviate unwanted emotions or induce positive sensations. Emotion regulation is also the most commonly reported motivation for self-harm across existing qualitative [13,14] and quantitative [16,81] reviews, and is a commonly identified self-harm function in existing theories of self-harm. For instance, the desire to control emotions by self-harm is consistent with the four-function model of NSSI which proposes that two functions of self-harm include automatic positive reinforcement (i.e., when emotions or sensations are generated through self-harm) and automatic negative reinforcement reasons (i.e., when self-harm removes unwanted thoughts or feelings) [82]. Both functions of self-harm seek to control emotions. In terms of using self-harm to control emotions, it is known that adolescence is an emotionally turbulent time, with the influence of shifting hormones and neurological development [83]. Evidence suggests that adolescents who struggle with unwanted emotions are more likely to experience worsening mental health outcomes in young adulthood [84]. Self-harm appears to be a means by which some young people seek to control their internal experience. Therapeutic interventions that support young people to tolerate these emotions, such as dialectical behaviour therapy (DBT) or acceptance and commitment therapy (ACT), may help to reduce self-harm incidents and improve the overall mental health of young people [85]. However, young people experience several barriers to accessing therapeutic intervention including poor availability of services and long waiting times [86], high cost of private mental healthcare, stigma around self-harm and privacy concerns (e.g., young people not wanting parents to be aware of the self-harm) [87]. Digital therapeutics have been posited as a potential solution to these barriers [88]. Additionally, given young peoples’ penchant for control, therapeutic interventions which are user led, such as digital therapeutics, may help provide users a greater sense of control [89], and may be more applicable for young people who are unwilling to disclose their self-harm to others [90].

The most common interpersonal motivation for self-harm was a desire to communicate with others to control how others respond to their distress. Studies reported that young people did not know how to share their distressed state with others and hoped that self-harm would be a means of receiving help. This is consistent with previous qualitative and quantitative reviews [13,14,16], and also consistent with the four-function model of NSSI [82]. In addition to positive and negative automatic reinforcement functions of self-harm, this theory also proposes that self-harm can occur for social positive reinforcement (i.e., when self-harm facilitates access to positive social outcomes, such as attention from others) and negative social reinforcement (i.e., when self-harm allows the individual to remove themselves from social situations or expectations) reasons. While these functions are not related to control of emotions, they are still linked to the concept of control in that they enable the individual to control their environment and interaction with others. Studies have posited that young people may have difficulty seeking help for mental health due to concerns about confidentiality being maintained, anticipated judgement, and stigma [90]. Worryingly, these concerns may even inhibit the disclosure of self-harm when the young person is already engaged with a psychologist [91]. Ensuring that young people, and those who support them such as peers, parents, teachers and health professionals, have adequate mental health literacy may improve disclosure of distress or self-harm and facilitate help-seeking [92].

This study has a number of strengths. First, all studies were independently double screened, increasing confidence that all relevant articles and findings have been included in this review. Furthermore, the rigorous and robust analysis process resulted in an in-depth synthesis of the included studies. This is also the first study to use Thematic Network Analysis [18] to identify an overarching theme to make sense of both precipitants and motivations of self-harm in young people. This interpretative approach to data synthesis allowed for deeper understanding of the self-harm precipitants and motivations. There are also a few limitations. Less than half of the included studies adequately described their participants or study settings, and less than half (43%) adequately reported on their data analysis process. Furthermore, only 15 (30%) studies were conducted in the global south, meaning the findings of this review may be less applicable to young people from such countries. Additionally, the qualitative analysis was undertaken by a female researcher from the global north, meaning interpretations of findings from the global south may not capture important cultural nuances. Furthermore, given that included studies were not restricted by origin, it is possible that the perception of control may differ across cultures (e.g., power differentials between males and females in certain cultures) and therefore the impact of control on self-harm may have significant cultural influences which we were unable to distinguish during this review but may be important when considering how to intervene or help young people who are self-harming. There were also few studies which examined the experience of self-harm for children (10–12 years), which is likely due to difficulties in undertaking sensitive research with this population. Nonetheless, this is an important area for future research given growing rates of self-harm in children [11]. Finally, only two studies conducted among LGBTQ+ young people, highlighting a need for additional research to be conducted on this population, particularly given that they are at heightened risk of suicide and self-harm [93].

## Conclusion

Overall, the current review identified an overarching influence of control in young peoples’ experience of self-harming behaviour. Young people are seeking to obtain control (motivation) in situations and environments in which they feel out of control (precipitant). When this control cannot be obtained or maintained, they resort to self-harm, a behaviour they can control. In order to address increasing rates of self-harm among young people, it may be helpful to provide young people with the knowledge and skills to manage their emotions and actions in uncertain situations. It may also be helpful to equip their network of family and friends with skills to appropriately support young people engaging in self-harm.

## Supporting information

S1 Checklist PRISMA Checklist. (DOCX)

S2 Appendix Search strategy. (DOCX)

S3 Appendix Quality assessment tool. (DOCX)

S4 Appendix Quality of included studies. (DOCX)

S5 File Reasons for exclusion during full-text screening. (DOCX)

S6 File Included and excluded studies from the final dataset. (DOCX)
