# Supplementary material for: The role of control in precipitating and motivating self-harm in young people: A systematic review and meta-synthesis of qualitative data
Source: PLoS One. 2025 Jun 13;20(6):e0325683. doi: 10.1371/journal.pone.0325683 (PMC12165347; doi:10.1371/journal.pone.0325683)
Supplement: S3 Appendix — (DOCX) [file pone.0325683.s003.docx]

**S3 Appendix. Quality assessment tool.**

| 1. **Were the criteria for inclusion in the sample clearly defined?** | |
| --- | --- |
| Adequate | The inclusion and exclusion criteria are clearly described. |
| Partial | Some information provided about the inclusion and exclusion criteria, but authors do not provide all necessary information critical to the study. |
| Poor/unclear | Insufficient information provided about the inclusion and exclusion criteria. |
| **2.** **Were the study subjects and setting described in detail?** | |
| Adequate | The authors provide an adequate description of study subjects and setting, including age, gender, and place and period of recruitment. |
| Partial | The authors provide some, but not full description of study subjects and setting. |
| Poor/unclear | The authors do not provide description of study subjects and setting, or have reported sample characteristics unclearly. |
| **3.** **Was the exposure (i.e., self-harm) measured in a valid and reliable way?** | |
| Adequate | Self-harm was clearly defined, and the method of measurement described in detail. The measurement of self-harm was accurate, reliable, and not prone to bias (i.e., a validated measure was used), or self-harm was ascertained based on hospital records (i.e., listed as a reason for admission to the service). |
| Partial | Authors have adapted a validated measure of self-harm, or have based self-harm on hospital records but have not provided sufficient detail about the part of the records they have based case selection on. |
| Poor/unclear | Measurement of self-harm is prone to bias. Measure(s) have poor reliability and/or validity, or authors have not used a validated measure of self-harm. Alternatively, authors have provided an insufficient description of how they measured/assessed self-harm. |
| **4.** **Was the outcome (e.g., function/motivation, precipitating circumstances) measured in a valid and reliable way?** | |
| Adequate | The method of measuring outcomes was described in detail. The measurement of outcomes was accurate, reliable, and not prone to bias (i.e., a validated measure was used). |
| Partial | Authors have adapted a validated measure of motivations/functions/precipitants/etc, or have used hospital records to determine outcomes. |
| Poor/unclear | Measurement of outcomes is prone to bias (e.g., based on medical records rather than a validated measure). Measure(s) have poor reliability and/or validity, or authors have provided an insufficient description of the measures used. |
| **5.** **Was appropriate statistical analysis used?** | |
| Adequate | The study clearly details the statistical analysis method used. The statistical analysis method is appropriate for the research question. |
| Partial | Statistical analysis method partly appropriate for the research question (e.g., statistical analysis does not account for multiple comparisons). |
| Poor/unclear | Insufficient description of statistical analysis method and/or inappropriate statistical analysis used to answer research questions. |
