# Supplementary material for: The role of control in precipitating and motivating self-harm in young people: A systematic review and meta-synthesis of qualitative data
Source: PLoS One. 2025 Jun 13;20(6):e0325683. doi: 10.1371/journal.pone.0325683 (PMC12165347; doi:10.1371/journal.pone.0325683)
Supplement: S4 Appendix — (DOCX) [file pone.0325683.s004.docx]

**S4 Appendix. Quality of included studies.**

|  | **Item 1. Inclusion criteria** | **Item 2. Study setting** | **Item 3. Appropriate methodology** | **Item 4.**  **Analytical rigour** | **Item 5. Appropriate conclusions** |
| --- | --- | --- | --- | --- | --- |
| Abeyaskekera & Marecek (2019) | + | / | - | / | / |
| Aggarwal et al. (2020) | / | / | + | / | / |
| Agüero et al. (2018) | + | + | - | - | / |
| Almeida et al. (2023) | + | / | + | + | + |
| Balaji et al. (2023) | + | + | + | + | + |
| Chen et al. (2021) | / | + | + | + | + |
| Cronemberger & de Silva (2023) | + | + | + | / | + |
| Curtis (2017) | - | / | - | - | / |
| Čuš et al. (2021) | + | + | + | + | + |
| Doyel et al. (2017) | + | / | / | - | / |
| Grandclerc et al. (2019) | + | + | + | / | + |
| Guest et al. (2021) | + | / | + | + | + |
| Gulbas et al. (2015) | - | / | + | / | + |
| Gulbas & Zayas (2015) | / | + | + | / | + |
| Hahm et al. (2014) | + | + | + | + | + |
| Hetrick et al. (2020) | + | + | + | / | + |
| Hird et al (2024) | + | / | + | + | + |
| Holliday & Vandermause (2015) | + | / | + | / | + |
| Holliday et al. (2020) | / | / | + | / | + |
| Latakiene & Skruibis (2015) | - | / | + | / | + |
| Lockwood et al. (2020) | - | - | + | + | + |
| Marzetti et al. (2023) | - | / | + | + | / |
| McAndrew & Warne (2014) | - | - | + | / | / |
| McClelland et al. (2022) | + | + | + | - | + |
| Miller et al. (2021) | + | / | + | / | + |
| Moraes et al. (2020) | + | + | / | / | + |
| Mughal et al. (2023) | + | + | + | / | + |
| Naz et al. (2021) | + | + | + | + | + |
| O’Brien et al. (2021) | / | + | + | + | + |
| Orri et al. (2014) | + | / | + | + | / |
| Quarshie et al. (2020) | - | / | + | + | + |
| Santo & Dell’Aglio | + | / | + | / | + |
| Shahwan et al. (2022) | + | + | + | / | + |
| Simões at al. (2021) | + | / | / | - | + |
| Sloan et al. (2021) | + | / | + | + | + |
| Stanicke et al. (2020) | + | / | + | + | + |
| Stanicke (2021) | + | + | + | + | + |
| Stradomska et al. (2016) | / | / | - | - | / |
| Sukhawaha et al. (2016) | + | + | + | / | + |
| Szlyk et al. (2019) | + | / | + | / | + |
| Taliaferro et al. (2019) | / | + | + | / | + |
| Tan et al. (2014) | + | / | + | / | / |
| Tan et al. (2019) | + | / | + | + | + |
| Tillman et al. (2018) | + | / | + | / | + |
| Tingey et al. (2014) | + | / | + | + | + |
| Wadman et al. (2017) | + | + | + | / | / |
| Wadman et al. (2018) | + | / | + | + | + |
| Williams et al. (2023) | - | + | + | / | + |
| Wong & Chung (2022) | + | / | + | + | + |
| Zhu et al. (2024) | + | + | + | + | + |
| % Adequate | 70% (n=35) | 42% (n=21) | 86% (n=43) | 42% (n=21) | 78% (n=39) |
| % Partial | 14% (n=7) | 54% (n=27) | 6% (n=3) | 46% (n=23) | 22% (n=11) |
| % Poor/unclear | 16% (n=8) | 4% (n=2) | 8% (n=4) | 12% (n=6) | 0% |

*Note.* N = 50. + Adequate; / Partial; - Poor/unclear.
