# Supplementary material for: The role of control in precipitating and motivating self-harm in young people: A systematic review and meta-synthesis of qualitative data
Source: PLoS One. 2025 Jun 13;20(6):e0325683. doi: 10.1371/journal.pone.0325683 (PMC12165347; doi:10.1371/journal.pone.0325683)
Supplement: S5 File — (DOCX) [file pone.0325683.s005.docx]

**S5 Supplementary Information. Reasons for exclusion during full-text screening.**

| **Author & year** | **Title** | **Screened by** | **Reason for exclusion** |
| --- | --- | --- | --- |
| Abhilash et al., 2022 | Changing trends in the profile of rodenticide poisoning | ST & AH | Outside age range/age not reported |
| Acherjya et al., 2020 | The scenario of acute poisoning in Jashore, Bangladesh | ST & AH | Outside age range/age not reported |
| Ahmad et al., 2017 | Pattern of suicide: A descriptive, comparative study conducted in Karachi during period 2011-2015 | AS & ST | Outside age range/age not reported |
| Akkaya-Kalayci et al., 2015 | The impact of migration and culture on suicide attempts of children and adolescents living in Istanbul | ST & WYZ | Does not examine topic of interest |
| Ali et al., 2022 | Racial/ethnic differences in preceding circumstances of suicide and potential suicide misclassification among US adolescents | ST & WYZ | Examines fatal suicide only |
| Andrews et al., 2013 | Predictors of continuation and cessation of nonsuicidal self-injury | ST & AH | Does not examine topic of interest |
| Aral et al., 2023 | The impact of dissociation and trauma on non-suicidal self-injury | AS & ST | Full text not available |
| Arens et al., 2014 | Child maltreatment and deliberate self-harm: a negative binomial hurdle model for explanatory constructs | ST & AH | Does not examine topic of interest |
| Ashworth et al., 2022 | Children and young people presenting in a pediatric emergency department in North-West England in suicidal crisis: An exploratory case series study | ST & AH | Analyses of interest not conducted exclusively in DSH sample |
| Ashworth et al., 2023 | Suicidal crisis among children and young people: Associations with adverse childhood experiences and socio-demographic factors | ST & HF | Does not examine topic of interest |
| Auerbach et al., 2014 | Adolescent nonsuicidal self-injury: examining the role of child abuse, comorbidity, and disinhibition | ST & AH | Does not examine topic of interest |
| Auerbach et al., 2017 | Impulsivity and suicidality in adolescent inpatients | ST & WYZ | Does not examine topic of interest |
| Babaeifard et al., 2024 | Early maladaptive schemas, distress tolerance and self-injury in Iranian adolescents: Serial mediation model of transdiagnostic factors | ST & AS | Does not examine topic of interest |
| Bai et al., 2024 | Influence of stress on self-injury among Chinese left-behind adolescents is not cast in stone: Synergistic roles of family protective factors | ST & AH | Does not examine topic of interest |
| Bareiss et al., 2014 | "Mauled by a Bear": narrative analysis of self-injury among adolescents in US news, 2007-2012 | ST & WYZ | Qualitative study without first-hand accounts |
| Bashir et al., 2014 | Deliberate self poisoning at national poisoning control centre | ST & AH | Outside age range/age not reported |
| Benarous et al., 2021 | Problematic substance use in adolescent psychiatric inpatients: rates, clinical correlates and effects on therapeutic outcomes | ST & WYZ | Does not examine topic of interest |
| Bennardi et al., 2016 | Risk of repeated self-harm and associated factors in children, adolescents and young adults | ST & AH | Does not examine topic of interest |
| Bethell et al., 2013 | Emergency department presentations for self-harm among Ontario youth | ST & AH | Does not examine topic of interest |
| Bhowmick et al., 2019 | A study on deliberately self-poisoned in-hospital patients in a tertiary health care center in northeast India: A cross-sectional review | ST & AH | Outside age range/age not reported |
| Bježančević et al., 2019 | Self-injury in adolescents: a five-year study of characteristics and trends | ST & AH | Does not examine topic of interest |
| Brady et al., 2014 | Cutting the silence: initial, impulsive self-cutting in adolescence | ST & AH | Not a study type of interest |
| Brausch et al., 2015 | Suicide-related concerns as a mediator between physical abuse and self-harm behaviors in college students | ST & WYZ | Does not examine topic of interest |
| Brausch et al., 2016 | Examining intent to die and methods for nonsuicidal self‐injury and suicide attempts | ST & AH | Does not examine topic of interest |
| Briones-Buixassa et al., 2021 | Predicting non-suicidal self-injury in young adults with and without borderline personality disorder: a multilevel approach combining ecological momentary assessment and self-report measures | ST & WYZ | Does not examine topic of interest |
| Brooks et al., 2015 | Understanding the social functions of nonsuicidal self-injury in community adolescents | ST & WYZ | Not a study type of interest |
| Brown et al., 2020 | "I just finally wanted to belong somewhere"-Qualitative analysis of experiences with posting pictures of self-Injury on Instagram | ST & AH | Does not examine topic of interest |
| Bryan et al., 2017 | Supporting LGBT Lives? Complicating the suicide consensus in LGBT mental health research | ST & WYZ | Outside age range/age not reported |
| Burke et al., 2016 | Identifying a physical indicator of suicide risk: Non-suicidal self-injury scars predict suicidal ideation and suicide attempts | ST & AH | Does not examine topic of interest |
| Cash et al., 2013 | Adolescent suicide statements on MySpace | ST & AH | Analyses of interest not conducted exclusively in DSH sample |
| Cerel et al., 2016 | Parents with suicidal behavior: Parenting is not always protective | ST & WYZ | Outside age range/age not reported |
| Chandler et al., 2021 | The violence of the cut: Gendering self-harm | ST & AH | Analyses of interest not conducted exclusively in DSH sample |
| Chartrand et al., 2015 | Correlates of nonsuicidal self-injury and suicide attempts among tertiary care, emergency department patients | ST & AH | Outside age range/age not reported |
| Chompoosri et al., 2021 | Sources of suicide methods: A survey in undergraduate students of a Northern Thai university with history of suicidal thoughts | ST & AH | Does not examine topic of interest |
| Choo et al., 2017 | What predicts medical lethality of suicide attempts in Asian youths? | ST & AH | Does not examine topic of interest |
| Cipriano et al., 2020 | Does anger expression mediate the relationship between parental rejection and direct and indirect forms of non-suicidal self-injury? | ST & AH | Does not examine topic of interest |
| Dayasiri et al., 2017 | Non-accidental poisoning among children in rural Sri Lanka: A two-year cross sectional study | ST & AS | Qualitative study without first-hand accounts |
| De Backer et al., 2024 | Women's experiences of attempted suicide in the perinatal period (ASPEN-study) - a qualitative study | ST & AS | Outside age range/age not reported |
| Devassy et al., 2020 | A cross-sectional study of the pattern of cases of deliberate self-harm in a tertiary care hospital | ST & AH | Outside age range/age not reported |
| Di Pierro et al., 2014 | Nonsuicidal self-injury as an affect-regulation strategy and the moderating role of impulsivity | ST & WYZ | Does not examine topic of interest |
| Dickstein et al., 2015 | Self-injurious implicit attitudes among adolescent suicide attempters versus those engaged in nonsuicidal self-injury | ST & WYZ | Does not examine topic of interest |
| Fallahi-Khoshknab et al., 2023 | Instability of emotional relationships and suicide among youth: a qualitative study | ST & WYZ | Outside age range/age not reported |
| Ferrara et al., 2019 | Pediatric suicide is a preventable phenomenon: A single center experience | ST & AH | Full text not available |
| Fitzgerald et al., 2017 | Non-suicidal self-injury in a New Zealand student population: Demographic and self-harm characteristics | ST & WYZ | Outside age range/age not reported |
| Fogaça et al., 2023 | Suicide attempts by adolescents assisted in an emergency department: a cross-sectional study | AS & AH | Does not examine topic of interest |
| Forbes et al., 2019 | Motives for nonsuicidal self-injury in individuals with lifetime depressive disorders and posttraumatic stress disorder | ST & AH | Outside age range/age not reported |
| Ghimire et al., 2014 | Psychiatric comorbidities in patients with deliberate self-harm in a tertiary care center | ST & AH | Outside age range/age not reported |
| Gouveia-Pereira et al., 2023 | Adolescents' perceptions about non-suicidal self-injury, suicidal ideation and suicide attempts | ST & AS | Analyses of interest not conducted exclusively in DSH sample |
| Grandclerc et al., 2017 | Study of specificities as regards the self-inflicted injuries experiences (non-suicidal self-injury and suicidal behavior) of adolescent girls born to migrant parents | ST & AH | Not in English language |
| Gray et al., 2023 | Why am I doing this? Ambivalence in the context of non-suicidal self-injury | ST & DR | Outside age range/age not reported |
| Haliczer et al., 2023 | Social stressors, emotional responses, and NSSI urges and behaviors in daily life | ST & AS | Does not examine topic of interest |
| Hansson et al., 2019 | A 15-year follow-up of former self-harming inpatients in child & adolescent psychiatry - a qualitative study | AS & AH | Outside age range/age not reported |
| Haroon et al., 2023 | Deliberate self harm - a hospital based cross sectional study | ST & AS | Outside age range/age not reported |
| Heesen et al., 2024 | The forever decision: a qualitative study among survivors of a suicide attempt | ST & AS | Outside age range/age not reported |
| Hill et al., 2023 | Cultural consensus modeling to identify culturally relevant reasons for and against suicide among Black adolescents | ST & AS | Does not examine self-harm/suicidal behaviours |
| Holley et al., 2015 | The lived experience of adolescents who engage in nonsuicidal self-injury | DR & BT | Not a study type of interest |
| Huey et al., 2014 | I had a lot of anger and that's what kind of led me to cutting myself': Employing a social stress framework to explain why some homeless women self-injure | ST & WYZ | Outside age range/age not reported |
| Hunt et al., 2020 | Experiences of suicide in transgender youth: A qualitative, community-based study | ST & WYZ | Analyses of interest not conducted exclusively in DSH sample |
| Idicula et al., 2019 | The emergency assessment and management of non-suicidal self-injury in adolescents | ST & WYZ | Not a study type of interest |
| Ingabire et al., 2020 | Suicidal ideation and behavior among Congolese refugees in Rwanda: Contributing factors, consequences, and support mechanisms in the context of culture | ST & AH | Outside age range/age not reported |
| Jackman et al., 2018 | Experiences of transmasculine spectrum people who report nonsuicidal self-injury: A qualitative investigation | ST & WYZ | Outside age range/age not reported |
| Jan et al., 2017 | Psychosocial risk factors and clinical profile associated with attempted suicide in young adult and adolescent patients in conflict zone-Kashmir | ST & WYZ | Does not examine topic of interest |
| Jegannathan et al., 2014 | 'Plue plun' male, 'kath klei' female: Gender differences in suicidal behavior as expressed by young people in Cambodia | ST & WYZ | Analyses of interest not conducted exclusively in DSH sample |
| Jørs et al., 2014 | Suicide attempts and suicides in Bolivia from 2007 to 2012: pesticides are the preferred method - females try but males commit suicide! | ST & AH | Analyses of interest not conducted exclusively in DSH sample |
| Karrouri et al., 2017 | Self-injury in hospitalized patients: Concerning 19 cases | ST & AH | Not in English language |
| Khan et al., 2013 | Pattern of poisoning in a tertiary level hospital | ST & AS | Full text not available |
| Kleiman et al., 2015 | Forms of non-suicidal self-injury as a function of trait aggression | ST & WYZ | Does not examine topic of interest |
| Krantz et al., 2022 | Fearlessness about death and suicide planning predict lethality of adolescent suicide attempts during and following treatment | ST & WYZ | Does not examine topic of interest |
| Kumar et al., 2017 | A study on clinical profile and trend in suicide attempters in psychiatry consultation | ST & AH | Outside age range/age not reported |
| Kumari et al., 2024 | Frequency and pattern of self-injurious behavior in children with intellectual disability presenting at a tertiary care hospital, Karachi Pakistan | AS & AH | Does not examine topic of interest |
| Lam et al., 2022 | "I thought that I had to be alive to repay my parents": Filial piety as a risk and protective factor for suicidal behavior in a qualitative study of Chinese women | ST & AH | Outside age range/age not reported |
| Lansing et al., 2017 | Assessing stress-related treatment needs among girls at risk for poor functional outcomes: The impact of cumulative adversity, criterion traumas, and non-criterion events | ST & AH | Does not examine topic of interest |
| Lear et al., 2019 | A daily diary investigation of the defective self model among college students with recent self-injury | ST & AH | Does not examine topic of interest |
| Lietzau et al., 2024 | Unwanted pursuit behavior victimization as a predictor of engagement in nonsuicidal self-injury | ST & AS | Full text not available |
| Lubbert et al., 2022 | Investigating the clinical profile of suicide attempters who used a violent suicidal means | ST & AH | Outside age range/age not reported |
| Marecek et al., 2023 | Gendered antecedents and consequences of young women's suicidal acts in Sri Lanka | ST & DR | Duplicate data |
| McPherson et al., 2022 | Teen advisory council survey's factors associated with self-harming thoughts | ST & AH | Does not examine self-harm/suicidal behaviours |
| Mellin et al., 2024 | A pilot study assessing sexual behavior as self-injury in college students | ST & AH | Does not examine topic of interest |
| Merza et al., 2017 | Characteristics and development of nonsuicidal super self-injury among borderline inpatients | ST & AH | Outside age range/age not reported |
| Mitchell et al., 2022 | Near-fatal self-harm among Canadian adolescents | ST & AH | Analyses of interest not conducted exclusively in DSH sample |
| Musyimi et al., 2020 | Suicidal behavior risks during adolescent pregnancy in a low-resource setting: A qualitative study | ST & AH | Analyses of interest not conducted exclusively in DSH sample |
| Pak et al., 2021 | Mixture modeling of nonsuicidal self-injury and binge eating: Behaviors and motives | ST & AS | Analyses of interest not conducted exclusively in DSH sample |
| Parajuli et al., 2024 | Belief and risk factors associated with suicidal and self-harm behaviour among young adults of Kathmandu district | ST & AS | Does not examine topic of interest |
| Paul et al., 2018 | Proximally-occurring life events and the first transition from suicidal ideation to suicide attempt in adolescents | ST & WYZ | Does not examine topic of interest |
| Piper et al., 2022 | A qualitative content analysis study using electronic medical records to understand youth suicide attempts | ST & AH | Qualitative study without first-hand accounts |
| Platt et al., 2024 | Suicide prevention programming across ecological levels: Recommendations from Latinx immigrant origin youth and their parents | ST & DR | Analyses of interest not conducted exclusively in DSH sample |
| Plener et al., 2016 | The prevalence of Nonsuicidal Self-Injury (NSSI) in a representative sample of the German population | ST & HF | Outside age range/age not reported |
| Price et al., 2019 | The changing characteristics of African-American adolescent suicides, 2001–2017 | ST & AH | Does not examine topic of interest |
| Pritchard et al., 2021* | Self-injury is my drug: the functions of describing nonsuicidal self-injury as an addiction | ST & WYZ | Outside age range/age not reported |
| Rawlings et al., 2015 | Out of the blue: Untangling the association between impulsivity and planning in self-harm | ST & WYZ | Does not examine topic of interest |
| Reangsing et al., 2024 | The experience of Thai adolescents with depression: A qualitative study | ST & DR | Does not examine self-harm/suicidal behaviours |
| Renymol & Suma, 2019 | Pattern of poisoning in a tertiary care center with special reference to odollam Poisoning | ST & AH | Outside age range/age not reported |
| Rissanen et al., 2013 | Factors helping adolescents to stop self-cutting: Descriptions of 347 adolescents aged 13-18 years | ST & AH | Does not examine topic of interest |
| Rivers et al., 2018 | LGBT people and suicidality in youth: A qualitative study of perceptions of risk and protective circumstances | ST & WYZ | Outside age range/age not reported |
| Robinson et al., 2023 | Communicating distress: suicide threats/gestures among clinical and community youth | ST & AS | Analyses of interest not conducted exclusively in DSH sample |
| Rogers et al., 2020 | Comparing suicide risk factors among individuals with a history of aborted, interrupted, and actual suicide attempts | ST & AH | Does not examine topic of interest |
| Rosario-Williams et al., 2022 | Factors precipitating suicide attempts vary across race | ST & AH | Outside age range/age not reported |
| Saarijärvi et al., 2023 | Early maladaptive schemas are associated with self-injury thoughts and behavior in adolescents | ST & AS | Analyses of interest not conducted exclusively in DSH sample |
| Sellers et al., 2021 | Nonsuicidal self-injury, suicide planning, and suicide attempts among high-risk adolescents prior to psychiatric hospitalization | ST & WYZ | Does not examine topic of interest |
| Simões et al., 2020 | Characterization of adopted suicidal behavior and its main influencing factors: A qualitative study with adolescents | ST & AH | Analyses of interest not conducted exclusively in DSH sample |
| Steinhoff et al., 2020 | Stressful life events in different social contexts are associated with self-injury from early adolescence to early adulthood | ST & WYZ | Does not examine topic of interest |
| Stroehmer et al., 2015 | Digital comparison of healthy young adults and borderline patients engaged in non-suicidal self-injury | ST & AH | Outside age range/age not reported |
| Tan et al., 2015 | "Nonsuicidal self-injury in an adolescent population in Singapore": Corrigendum | ST & AH | Not a study type of interest |
| Tassi et al., 2018 | Callous-unemotional traits, borderline personality, and self-injury in gothic subculture | ST & AH | Outside age range/age not reported |
| Tharani et al., 2022 | Characteristics and patterns of individuals who have self-harmed: a retrospective descriptive study from Karachi, Pakistan | ST & AH | Outside age range/age not reported |
| Troya et al., 2021* | Investigating the relationship between childhood sexual abuse, self-harm repetition and suicidal intent: Mixed-methods study | ST & AH | Outside age range/age not reported |
| Trujillo & Servaty-Seib, 2018 | Parental absence and non-suicidal self-injury: Social support, social constraints and sense-making | ST & WYZ | Outside age range/age not reported |
| Tsypes et al., 2016 | Non-suicidal self-injury and suicidal thoughts and behaviors in heterosexual and sexual minority young adults | ST & AH | Does not examine topic of interest |
| Van Alphen et al., 2017 | Predictors of rehospitalization for depressed adolescents admitted to acute psychiatric treatment | ST & WYZ | Does not examine topic of interest |
| Victor et al., 2019 | I want you to want me: Interpersonal stress and affective experiences as within‐person predictors of nonsuicidal self‐injury and suicide urges in daily life | ST & WYZ | Does not examine self-harm/suicidal behaviours |
| Vivier et al., 2021 | Suicidal behaviors in migrant youths: Male experiences | ST & AH | Not in English language |
| Vuscan et al., 2022 | Risk factors for suicidal behavior: A Romanian mixed methods study | ST & AH | Outside age range/age not reported |
| Walls et al., 2014 | "Rebuilding our community": hearing silenced voices on Aboriginal youth suicide | ST & AH | Qualitative study without first-hand accounts |
| Wang et al., 2023 | Impact of the COVID-19 and psychological risk factors on non-suicidal self-injury behavior among high school students: a one-year follow-up study | ST & AS | Does not examine topic of interest |
| Wang et al., 2021 | Functional assessment of restrictive eating: A three-study clinically heterogeneous and transdiagnostic investigation | ST & AH | Analyses of interest not conducted exclusively in DSH sample |
| Wei et al., 2017 | Comparison of the characteristics of suicide attempters with major depressive disorder and those with no psychiatric diagnosis in emergency departments of general hospitals in China | ST & AH | Outside age range/age not reported |
| Whitlock et al., 2015 | Predictors of self-injury cessation and subsequent psychological growth: Results of a probability sample survey of students in eight universities and colleges | ST & WYZ | Does not examine topic of interest |
| Wu et al., 2021 | A large sample survey of suicide risk among university students in China | ST & AH | Analyses of interest not conducted exclusively in DSH sample |
| Young et al., 2017 | Closing in on crisis: informing clinical practice regarding nonsuicidal self-injury in youth | ST & DR | Qualitative study without first-hand accounts |
| Yu et al., 2024 | Non-suicidal self-injury motivation scale in a community sample of adolescents: a methodological study | ST & AS | Does not examine topic of interest |
| Zortea et al., 2019 | Associations between experiences of disrupted attachments and suicidal thoughts and behaviours: An interpretative phenomenological analysis | ST & AH | Outside age range/age not reported |
| Zungu et al., 2022 | 'They sent me out to school, and I came back with a baby': Perinatal women's experiences of biographical disruption and nonfatal suicidal behaviour | ST & AH | Outside age range/age not reported |

*These articles appeared twice during the full-text screening stage as they were not identified as duplicates during the duplicate removal process. DSH = deliberate self-harm.
