# Supplementary material for: The role of control in precipitating and motivating self-harm in young people: A systematic review and meta-synthesis of qualitative data
Source: PLoS One. 2025 Jun 13;20(6):e0325683. doi: 10.1371/journal.pone.0325683 (PMC12165347; doi:10.1371/journal.pone.0325683)
Supplement: S6 File — (DOCX) [file pone.0325683.s006.docx]

**S6 Supplementary Information. Included and excluded studies from the final dataset.**

| **Study ID** | **Title** | **Extractors** | **Decision** |
| --- | --- | --- | --- |
| Abbasian et al., 2021 | The role of psychological factors in non-suicidal self-injury of female adolescents | ST & AH | Exclude - not qualitative |
| Abeyasekera et al., 2019 | Embodied shame and gendered demeanours in young women in Sri Lanka | DR & AH | Include |
| Aggarwal et al., 2020 | Explanatory style in youth self-harm: an Indian qualitative study to inform intervention design | DR & AH | Include |
| Agüero et al., 2018 | Self-injurious behaviors among adolescents: A qualitative study of characteristics, meanings, and contexts | ST & DR | Include |
| Akkaya-Kalayci et al., 2018 | Triggers for attempted suicide in Istanbul youth, with special reference to their socio-demographic background | ST &AH | Exclude - not qualitative |
| Almeida et al., 2023 | "In the moment I wanted to kill myself, and then after I didn't": A qualitative investigation of suicide planning, method choice, and method substitution among adolescents hospitalized following a suicide attempt | DR & AH | Include |
| Ammerman et al., 2021 | Characterizing the choice to disclose nonsuicidal self-injury | AH & BT | Exclude - not qualitative |
| Andrei et al., 2024 | Non-suicidal self-injury (NSSI) patterns in adolescents from a Romanian child psychiatry inpatient clinic | ST & AH | Exclude - not qualitative |
| Andrewes et al., 2017 | Ecological momentary assessment of nonsuicidal self-injury in youth with borderline personality disorder | AH & BT | Exclude - not qualitative |
| Armiento et al., 2014 | An examination of disclosure of nonsuicidal self-injury among university students | AH & GH | Exclude - not qualitative |
| BabcockFenerci et al., 2022 | Maltreatment history and reasons for self-injurious behavior among adolescents engaged in non-suicidal self-injury versus adolescents who attempted suicide | ST & AH | Exclude - not qualitative |
| Bahali et al., 2024 | Parental attachment and childhood trauma in adolescents engaged in non-suicidal self-injury | AH & GH | Exclude - not qualitative |
| Balaji et al., 2023 | Why young people attempt suicide in India: A qualitative study of vulnerability to action | DR & AH | Include |
| BarretoCarvalho et al., 2017 | Biting myself so I don't bite the dust: prevalence and predictors of deliberate self-harm and suicide ideation in Azorean youths | ST & GH | Exclude - not qualitative |
| Batejan et al., 2015 | Perceptions of the functions of nonsuicidal self-injury in a college sample | ST & BT | Exclude - not qualitative |
| Beckman et al., 2019 | Impulsive suicide attempts among young people-A prospective multicentre cohort study in Sweden | ST & AH | Exclude - not qualitative |
| Bentley et al., 2015 | The unique contributions of distinct experiential avoidance domains to severity and functionality of non-suicidal self-injury | ST & AS | Exclude - not qualitative |
| Bhanuprakash et al., 2023 | Profile of acute poisoning in adolescents from Southern India: A prospective cohort study | ST & AH | Exclude - not qualitative |
| Bhola et al., 2017 | Predictors of non-suicidal and suicidal self-injurious behaviours, among adolescents and young adults in urban India | ST & GH | Exclude - not qualitative |
| Bilginer et al., 2017 | Overview of young people attempting suicide by drug overdose and prevention and protection services | ST & AH | Exclude - not qualitative |
| Braga et al., 2014 | Non-suicidal self injury, psychopathology and attachment: a study with university students | ST & BT | Exclude - not qualitative |
| Brausch et al., 2016 | Nonsuicidal self-injury disorder: Does Criterion B add diagnostic utility? | ST & BT | Exclude - not qualitative |
| Brausch et al., 2018 | Perceived effectiveness of NSSI in achieving functions on severity and suicide risk | ST & AH | Exclude - not qualitative |
| Burón et al., 2016 | Reasons for attempted suicide in Europe: prevalence, associated factors, and risk of repetition | ST & AH | Exclude - not qualitative |
| Calvete et al., 2015 | Prevalence and functions of non-suicidal self-injury in Spanish adolescents | ST & BT | Exclude - not qualitative |
| Carranza et al., 2022 | Nonsuicidal self-injury and intimate partner violence: Directionality of violence and motives for self-injury | AH & GH | Exclude - not qualitative |
| Carvalho et al., 2023 | The enhancing role of emotion regulation in the links between early positive memories and self-harm and suicidal ideation in adolescence | ST & AH | Exclude - not qualitative |
| Carvalho et al., 2023 | Emotion regulation weakens the associations between parental antipathy and neglect and self-harm | AH & AS | Exclude - not qualitative |
| Case et al., 2020 | Functions of non-suicidal self-injury in late adolescence: A latent class analysis | ST & AH | Exclude - not qualitative |
| Chen et al., 2021 | A qualitative study of how self-harm starts and continues among Chinese adolescents | DR & BT | Include |
| Chen et al., 2024 | The characteristics of suicide attempters in Chinese patients: Comparison of adolescents, youths, middle-aged and elderly | ST & AH | Exclude - not qualitative |
| Choo et al., 2018 | Suicide precipitants differ across the lifespan but are not significant in predicting medically severe attempts | ST & BT | Exclude - not qualitative |
| Christoforou et al., 2021 | Emotion profiles of university students engaging in non-suicidal self-injury: Association with functions of self-injury and other mental health concerns | ST & AH | Exclude - not qualitative |
| Coppersmith et al., 2021 | Variability in the functions of nonsuicidal self-injury: evidence from three real-time monitoring studies | AS & GH | Exclude - not qualitative |
| Costa et al., 2021 | Profile of non-suicidal self-injury in adolescents: interface with impulsiveness and loneliness | ST & AH | Exclude - not qualitative |
| Cronemberger et al., 2023 | Nonsuicidal self-injury in young women: understanding the meanings involved in the self-injurious act | DR & AH | Include |
| Curtis et al., 2017 | Non-suicidal self-injury: Suicide risk or social activity? | DR & BT | Include |
| Čuš et al., 2021 | “Smartphone apps are cool, but do they help me?”: A qualitative interview study of adolescents' perspectives on using smartphone interventions to manage nonsuicidal self-injury | DR & BT | Include |
| Czyz et al., 2019 | Daily patterns in nonsuicidal self-injury and coping among recently hospitalized youth at risk for suicide | ST & AH | Exclude - not qualitative |
| Czyz et al., 2021 | Short-term associations between nonsuicidal and suicidal thoughts and behaviors: A daily diary study with high-risk adolescents | DR & AH | Exclude - not qualitative |
| DiCorcia et al., 2017 | Methods and functions of non-suicidal self-injury among adolescents seeking emergency psychiatric services | ST & AH | Exclude - not qualitative |
| Dixon-Gordon et al., 2022 | Self-injury motives: A person-centered examination | ST & AH | Exclude - not qualitative |
| Doyle et al., 2017 | Motivations for adolescent self-harm and the implications for mental health nurses | ST & DR | Include |
| Doyle et al., 2017 | Attitudes toward adolescent self-harm and its prevention: The views of those who self-harm and their peers | AH & BT | Exclude - mixed methods, but qualitative data does not address research question |
| Duarte et al., 2019 | Social representations about the functions of deliberate self-harm: Adolescents and parents | ST & AS | Exclude - not qualitative |
| Duarte et al., 2020 | How do families represent the functions of deliberate self-harm? A comparison between the social representations from adolescents and their parents | ST & AS | Exclude - not qualitative |
| Eskin et al., 2019 | Suicidal thoughts, attempts and motives among university students in 12 Muslim-majority countries | ST & DR | Exclude - not qualitative |
| Eskin et al., 2022 | Are nonfatal suicide attempts instrumental in achieving personal and interpersonal goals? | ST & GH | Exclude - not qualitative |
| Faura-García et al., 2022 | Nonsuicidal self-injury thoughts and behavior in adolescents: Validation of SITBI-NSSI | ST & AH | Exclude - not qualitative |
| Gandhi et al., 2016 | Sociotropy, autonomy, and non-suicidal self-injury: The mediating role of identity confusion | ST & AS | Exclude - not qualitative |
| Gandhi et al., 2021 | Non-suicidal self-injury and its association with identity formation in India and Belgium: A cross-cultural case-control study | ST & AH | Exclude - not qualitative |
| Garcia-Nieto et al., 2015 | Clinical correlates of non-suicidal self-injury (NSSI) in an outpatient sample of adolescents | ST & BT | Exclude - not qualitative |
| Gardner et al., 2020 | The significance of site of cut in self-harm in young people | AH & BT | Exclude - not qualitative |
| Gardner et al., 2021 | Intrapersonal and interpersonal functions as pathways to future self-harm repetition and suicide attempts | ST & GH | Exclude - not qualitative |
| Gatta et al., 2022 | Hospitalisation in child neuropsychiatry: a case study along a five-year epidemiological-clinical trend | ST & BT | Exclude - not qualitative |
| Gholamrezaei et al., 2023 | Function of nonsuicidal self-injury among Iranian university students: a factor structure analysis | ST & AH | Exclude - not qualitative |
| Goddard et al., 2021 | Big five personality clusters in relation to nonsuicidal self-injury | ST & GH | Exclude - not qualitative |
| Grandclerc et al., 2019 | The quest for meaning around self-injurious and suicidal acts: A qualitative study among adolescent girls | DR & AH | Include |
| Gray et al., 2022 | Cognitive and emotional factors associated with the desire to cease non-suicidal self-injury | GH & BT | Exclude - not qualitative |
| Groschwitz et al., 2015 | The association of non-suicidal self-injury and suicidal behavior according to DSM-5 in adolescent psychiatric inpatients | ST & BT | Exclude - not qualitative |
| Grudnikoff et al., 2015 | Suicidality and hospitalization as cause and outcome of pediatric psychiatric emergency room visits | ST & BT | Exclude - not qualitative |
| Guérin-Marion et al., 2021 | Profiles of emotion dysregulation among university students who self-injure: Associations with parent–child relationships and non-suicidal self-injury characteristics | ST & AH | Exclude - not qualitative |
| Guan et al., 2024 | The impact of depressive and anxious symptoms on non-suicidal self-injury behavior in adolescents: a network analysis | ST & GH | Exclude - not qualitative |
| Guest et al., 2021 | A qualitative exploration of young people's experiences of attempted suicide in the context of alcohol and substance use | DR & BT | Include |
| Gulbas et al., 2015 | Examining the interplay among family, culture, and latina teen suicidal behavior | DR & GH | Include |
| Gulbas et al., 2015 | An exploratory study of nonsuicidal self-injury and suicidal behaviors in adolescent Latinas | DR & GH | Include |
| Gungordu et al., 2024 | Peer bullying and psychiatric diagnoses in adolescents with nonsuicidal self-injury | ST & WYZ | Exclude - not qualitative |
| Hahm et al., 2014 | Fractured identity: A framework for understanding young Asian American women's self-harm and suicidal behaviors | DR & AH | Include |
| Halder et al., 2016 | Socio-demographic and clinical characteristics of patients who attempt suicide: A hospitalbased study from eastern India | ST & BT | Exclude - not qualitative |
| Hamza et al., 2014 | A laboratory examination of pain threshold and tolerance among nonsuicidal self-injurers with and without self-punishing motivations | GH & BT | Exclude - not qualitative |
| Hetrick et al., 2020 | Understanding the needs of young people who engage in self-harm: A qualitative investigation | ST & DR | Include |
| Hettiarachchi et al., 2018 | Self-harm among young people detained in the youth justice system in Sri Lanka | DR & AH | Exclude - not qualitative |
| Hird et al., 2024 | Trans young people's experiences of nonsuicidal self-injury | DR & BT | Include |
| Hiremath et al., 2016 | Adolescent deliberate self-poisoning in South-East Melbourne | ST & BT | Exclude - not qualitative |
| Holliday et al., 2015 | Teen experiences following a suicide attempt | DR & AH | Include |
| Holliday et al., 2020 | Understanding adolescents' experiences of self-harm: Secondary analysis of family therapy sessions from the SHIFT trial | DR & AH | Include |
| Horowitz et al., 2018 | The relationship between interpersonal trauma history and the functions of non-suicidal self-injury in young adults: An experience sampling study | ST & AH | Exclude - not qualitative |
| Idig-Camuroglu et al., 2018 | Non-suicidal self-injury among university students in Turkey: the effect of gender and childhood abuse | AH & BT | Exclude - not qualitative |
| Ilieff et al., 2023 | A longitudinal examination of recent posttraumatic stress symptoms and nonsuicidal self-injury among university students | ST & GH | Exclude - not qualitative |
| Jacobson 2013 | Reasons for attempting suicide among a community sample of adolescents | ST & GH | Exclude - not qualitative |
| Jiang et al., 2022 | The relationship between negative life events and non-suicidal self-injury (NSSI) among Chinese junior high school students: the mediating role of emotions | ST & BT | Exclude - not qualitative |
| Jonsson et al., 2019 | Similarities and differences in the functions of nonsuicidal self‐injury (NSSI) and sex as self‐injury (SASI) | ST & AH | Exclude - not qualitative |
| Kądziela-Olech et al., 2015 | The prevalence of Non-suicidal Self-Injury (NSSI) among high school students in relation to age and sex | ST & AH | Exclude - not qualitative |
| Kaess et al., 2013 | Adverse childhood experiences and their impact on frequency, severity, and the individual function of nonsuicidal self-injury in youth | ST & AH | Exclude - not qualitative |
| Kandsperger et al., 2022 | Emotional reactivity in adolescents with non-suicidal self-injury and its predictors: a longitudinal study | ST & AH | Exclude - not qualitative |
| Ke et al., 2024 | Childhood maltreatment and engaging in NSSI for automatic-negative reinforcement: The mediating role of alexithymia and moderating role of help-seeking attitudes | ST & GH | Exclude - not qualitative |
| Kharsati et al., 2015 | Patterns of non-suicidal self-injurious behaviours among college students in India | ST & AH | Exclude - not qualitative |
| Khutoryanskaya et al., 2023 | Nonsuicidal self-injury behavior in adolescents | ST & BT | Exclude - not qualitative |
| Kiekens et al., 2017 | What predicts ongoing nonsuicidal self-injury?: A comparison between persistent and ceased self-injury in emerging adults | ST & GH | Exclude - not qualitative |
| Kim et al., 2021 | Cost-effectiveness of a multi-disciplinary emergency consultation system for suicide attempts by drug overdose in young people and adult populations | GH & BT | Exclude - not qualitative |
| Kim et al., 2022 | Changes in suicide rate and characteristics according to age of suicide attempters before and after COVID-19 | GH & BT | Exclude - not qualitative |
| King et al., 2024 | 24-Hour warning signs for adolescent suicide attempts | ST & AS | Exclude - not qualitative |
| Klonsky et al., 2015 | The functions of nonsuicidal self-injury: Converging evidence for a two-factor structure | ST & GH | Exclude - not qualitative |
| Knorr et al., 2013 | The role of sensation seeking in Non-suicidal self-injury | ST & AH | Exclude - not qualitative |
| Kostic et al., 2019 | Nonsuicidal self-injury among adolescents in south-east Serbia | ST & AH | Exclude - not qualitative |
| Kostic et al., 2024 | Nonsuicidal self-injury and suicidal beliefs in adolescent inpatient | ST & GH | Exclude - not qualitative |
| Kraus et al., 2020 | Anti-Suicide Function of Nonsuicidal Self-Injury in Female Inpatient Adolescents | ST & AH | Exclude - not qualitative |
| Kwan et al., 2021 | Does personality problems increase youth suicide risk?: A characteristic analysis study of youth who visit the emergency department following suicide attempt | ST & GH | Exclude - not qualitative |
| Latakiene et al., 2015 | Attempted suicide: Qualitative study of adolescent females' lived experience | ST & DR | Include |
| Lee et al., 2016 | Psychological characteristics of self-harming behavior in Korean adolescents | ST & GH | Exclude - not qualitative |
| Lee et al., 2019 | Characteristics of adolescents who visit the emergency department following suicide attempts: Comparison study between adolescents and adults | ST & GH | Exclude - not qualitative |
| Lockwood et al., 2020 | What young people say about impulsivity in the short-term build up to self-harm: A qualitative study using card-sort tasks | DR & BT | Include |
| Lockwood et al., 2023 | A comparison of temporal pathways to self-harm in young people compared to adults: A pilot test of the Card Sort Task for Self-harm online using Indicator Wave Analysis | AS & GH | Exclude - not qualitative |
| Luo et al., 2024 | Functions of nonsuicidal self-injury and repeated nonsuicidal self-injury among adolescents: A moderating role of addictive features | ST & BT | Exclude - not qualitative |
| Luyckx et al., 2015 | Non-suicidal self-injury in female adolescents and psychiatric patients: A replication and extension of the role of identity formation | ST & BT | Exclude - not qualitative |
| Lyons et al., 2021 | Life experiences preceding high lethality suicide attempts in adolescents at a level I regional trauma center | ST & AH | Exclude - not qualitative |
| Ma et al., 2023 | Current status of nonsuicidal injuries and associated factors among junior high school students in Hainan Province, China: a cross-sectional study | ST & BT | Exclude - not qualitative |
| Mahtani et al., 2018 | Shame proneness, shame coping, and functions of nonsuicidal self-injury (NSSI) among emerging adults: A developmental analysis | ST & AH | Exclude - not qualitative |
| Marzetti et al., 2023 | A qualitative study of young people's lived experiences of suicide and self-harm: intentionality, rationality and authenticity | ST & DR | Include |
| May et al., 2016 | Descriptive and psychometric properties of the inventory of motivations for suicide attempts (IMSA) in an inpatient adolescent sample | ST & BT | Exclude - not qualitative |
| McAndrew et al., 2014 | Hearing the voices of young people who selfâ€harm: Implications for service providers | DR & BT | Include |
| McClelland et al., 2022 | A Qualitative Exploration of the Experiences and Perceptions of Interpersonal Relationships Prior to Attempting Suicide in Young Adults | ST & DR | Include |
| McManus et al., 2019 | Prevalence of non-suicidal self-harm and service contact in England, 2000-14: repeated cross-sectional surveys of the general population | ST & GH | Exclude - not qualitative |
| Mehmood et al., 2023 | Prevalence of non-suicidal self-injury in medical students of Rawalpindi; its socio-demographics, methods, and functions | ST & GH | Exclude - not qualitative |
| Miller et al., 2021 | A qualitative study of understanding reasons for self-harm in adolescent girls | ST & DR | Include |
| Mirichlis et al., 2022 | Correlates of disclosure of non-suicidal self-injury amongst Australian university students | ST & DR | Exclude - not qualitative |
| Moraes et al., 2020 | "The pen is the blade, my skin the paper": risk factors for self-injury in adolescents | ST & DR | Include |
| Mroczkowska-Juchkiewicz et al., 2016 | Intentional poisonings in urban and rural children - a 6-year retrospective single centre study | ST & AH | Exclude - not qualitative |
| Muehlenkamp et al., 2013 | Interpersonal features and functions of nonsuicidal self-injury | ST & GH | Exclude - not qualitative |
| Mughal et al., 2023 | The functions of self-harm in young people and their perspectives about future general practitioner-led care: A qualitative study | ST & DR | Include |
| Nagy et al., 2024 | Negative self-perceptions and severity of NSSI: Testing the benefits and barriers model | ST & GH | Exclude - not qualitative |
| Naz et al., 2021 | Exploring lived experiences of adolescents presenting with self-harm and their views about suicide prevention strategies: a qualitative approach | DR & BT | Include |
| Nicol et al., 2022 | The relationship between early maladaptive schemas and the functions of self-injurious behaviour in youth | ST & WYZ | Exclude - not qualitative |
| O'Brien et al., 2021 | Why adolescents attempt suicide: a qualitative study of the transition from ideation to action | DR & BT | Include |
| Ong et al., 2017 | Functions of nonsuicidal self-injury in Singapore adolescents: Implications for intervention | ST & DR | Exclude - not qualitative |
| Orri et al., 2014 | Qualitative approach to attempted suicide by adolescents and young adults: the (neglected) role of revenge | ST & DR | Include |
| Özlü-Erkilic et al., 2020 | Transcultural differences in risk factors and in triggering reasons of suicidal and self-harming behaviour in young people with and without a migration background | ST & BT | Exclude - not qualitative |
| Pérez Rodríguez et al., 2021 | Characteristics and unidimensionality of non-suicidal self-injury in a community sample of spanish adolescents | ST & GH | Exclude - not qualitative |
| Park et al., 2015 | Clinical characteristics and precipitating factors of adolescent suicide attempters admitted for psychiatric inpatient care in south Korea | ST & GH | Exclude - not qualitative |
| Park et al., 2022 | Association of socioeconomic status with nonsuicidal self-injury and suicidal ideation in young Korean adults | ST & GH | Exclude - not qualitative |
| Park et al., 2024 | Characteristics and functions of non-suicidal self-injury that inform suicide risk | ST & GH | Exclude - not qualitative |
| Paul et al., 2015 | Frequency and functions of non-suicidal self-injury: associations with suicidal thoughts and behaviors | ST & GH | Exclude - not qualitative |
| Peters et al., 2019 | Sexual orientation differences in non-suicidal self-injury, suicidality, and psychosocial factors among an inpatient psychiatric sample of adolescents | ST & BT | Exclude - not qualitative |
| Piarulli et al., 2023 | Do cortisol and dehydroepiandrosterone influence motivational factors for non-suicidal self-injury in female adolescents? | ST & GH | Exclude - not qualitative |
| Pollak et al., 2020 | Does function predict persistence? Nonsuicidal self-injury among adolescents during and after hospitalization | ST & BT | Exclude - not qualitative |
| Quarshie et al., 2020 | Adolescent self-harm in Ghana: a qualitative interview-based study of first-hand accounts | ST & DR | Include |
| Radziwiłłowicz & Lewandowska, 2017 | Deliberate self-injury functions and their clinical correlates among adolescent psychiatric inpatients | ST & GH | Exclude - not qualitative |
| Rajapakse et al., 2015 | Non-fatal self-poisoning in Sri Lanka: associated triggers and motivations | ST & DR | Exclude - not qualitative |
| Rasmussen et al., 2016 | Why do adolescents self-harm? An investigation of motives in a community sample | ST & GH | Exclude - not qualitative |
| Reinhardt et al., 2021 | Functions of nonsuicidal self-injury in a Hungarian community adolescent sample: a psychometric investigation | ST & AS | Exclude - not qualitative |
| Reinhardt et al., 2021 | Latent class analysis of nonsuicidal self-injury among justice-involved juveniles: Association with motivational and emotional aspects of self-harm behavior | ST & AS | Exclude - not qualitative |
| Reinhardt et al., 2022 | Non-suicidal self-injury motivations in the light of self-harm severity indicators and psychopathology in a clinical adolescent sample | ST & GH | Exclude - not qualitative |
| Reinhardt et al., 2022 | A person-centered approach to adolescent nonsuicidal self-injury: Predictors and correlates in a community sample | ST & GH | Exclude - not qualitative |
| Robillard et al., 2022 | Support for a transdiagnostic motivational model of self-damaging behaviors: comparing the salience of motives for binge drinking, disordered eating, and nonsuicidal self-injury | ST & GH | Exclude - not qualitative |
| Robinson et al., 2021 | Nonsuicidal self-injury thoughts and behavioural characteristics: Associations with suicidal thoughts and behaviours among community adolescents | ST & BT | Exclude - not qualitative |
| Rodav et al., 2014 | Clinical characteristics and functions of non-suicide self-injury in youth | ST & GH | Exclude - not qualitative |
| Roley-Roberts et al., 2017 | Functions of nonsuicidal self‐injury are differentially associated with suicide ideation and past attempts among childhood trauma survivors | ST & GH | Exclude - not qualitative |
| Sack et al., 2022 | Typologies of non-suicidal self-injury functions and clinical correlates among inpatient youth | ST & GH | Exclude - not qualitative |
| Sack et al., 2024 | Typologies of non-suicidal self-injury functions and clinical correlates among inpatient youth | ST & GH | Exclude - duplicate |
| Sadeh et al., 2014 | Functions of non-suicidal self-injury in adolescents and young adults with Borderline Personality Disorder symptoms | ST & BT | Exclude - not qualitative |
| Santo et al., 2022 | Self-injury in adolescence from the bioecological perspective of human development | DR & GH | Include |
| Saraff et al., 2014 | Functions, lifetime frequency, and variety of methods of non-suicidal self-injury among college students | DR & BT | Exclude - not qualitative |
| Saraff et al., 2015 | Functions, Consequences, and Frequency of Non-suicidal Self-Injury | ST & GH | Exclude - not qualitative |
| Schmidt et al., 2023 | Non-suicidal self-injury in young adults with and without borderline personality disorder: The role of emotion dysregulation and negative urgency | ST & AH | Exclude - not qualitative |
| Selby et al., 2014 | How does self-injury feel? Examining automatic positive reinforcement in adolescent self-injurers with experience sampling | ST & BT | Exclude - not qualitative |
| Shahwan et al., 2018 | Deliberate self-harm in psychiatric outpatients aged 14-35 years in Singapore | ST & BT | Exclude - not qualitative |
| Shahwan et al., 2020 | A typology of nonsuicidal self-injury in a clinical sample: A latent class analysis | ST & AS | Exclude - not qualitative |
| Shahwan et al., 2022 | A qualitative study of motivations for non-suicidal self-injury in a sample of psychiatric outpatients in Singapore | DR & BT | Include |
| Shen et al., 2023 | Non-suicidal self-injury function: prevalence in adolescents with depression and its associations with non-suicidal self-injury severity, duration and suicide | ST & GH | Exclude - not qualitative |
| Shi et al., 2023 | Early maladaptive schemas and the risk of nonsuicidal self-injury in college students: A retrospective study | ST & BT | Exclude - not qualitative |
| Shingleton et al., 2013 | Binge/purge thoughts in nonsuicidal self-injurious adolescents: an ecological momentary analysis | ST & AS | Exclude - not qualitative |
| Silverman et al., 2018 | Non-suicidal self-injury among male adjudicated adolescents: Psychosocial concerns, coping responses, diagnoses, and functions | ST & BT | Exclude - not qualitative |
| Simões et al., 2021 | Reasons assigned to suicide attempts: adolescents' perceptions | ST & DR | Include |
| Singhal et al., 2021 | Non-suicidal self-injury (NSSI) among emerging adults: Sub-group profiles and their clinical relevance | ST & BT | Exclude - not qualitative |
| Sloan et al., 2021 | A qualitative examination of the relationship between rumination, distress, and dysregulated behaviours in vulnerable young people | ST & DR | Include |
| Stacy et al., 2018 | Before, during, and after self injury: The practice patterns of nonsuicidal self injury | ST & AS | Exclude - not qualitative |
| Stanicke et al., 2020 | Discovering one's own way: Adolescent girls' different pathways into and out of self-harm | ST & DR | Include |
| Stanicke et al., 2021 | The punished self, the unknown self, and the harmed self-Toward a more nuanced understanding of self-harm among adolescent girls | ST & DR | Include |
| Stewart et al., 2017 | Adolescent self-injurers: Comparing non-ideators, suicide ideators, and suicide attempters | ST & BT | Exclude - not qualitative |
| Stradomska et al., 2016 | Circumstances and underlying causes of suicidal attempts in teen patients of mental health facilities - A psychological perspective | ST & AS | Include |
| Sukhawaha et al., 2016 | Attempted suicide triggers in Thai adolescent perspectives | DR & BT | Include |
| Szewczuk-Boguslawska et al., 2021 | Self-injuries and their functions with respect to suicide risk in adolescents with conduct disorder: Findings from a path analysis | ST & AS | Exclude - not qualitative |
| Szlyk et al., 2019 | “I just kept it to myself”: The shaping of Latina suicidality through gendered oppression, silence, and violence | DR & BT | Include |
| Taş Torun et al., 2022 | Intra/interpersonal functions of non-suicidal self-injury in adolescents with major depressive disorder: the role of emotion regulation, alexithymia, and childhood traumas | ST & AS | Exclude - not qualitative |
| Taliaferro et al., 2019 | Function and progression of non-suicidal self-injury and relationship with suicide attempts: A qualitative investigation with an adolescent clinical sample | DR & BT | Include |
| Tan et al., 2014 | Nonsuicidal self-injury in an adolescent population in Singapore | ST & DR | Include |
| Tan et al., 2019 | Feeling better or worse? The lived experience of non-suicidal self-injury among Malaysian university students | DR & BT | Include |
| Thai et al., 2021 | The prevalence, correlates and functions of non-suicidal self-injury in Vietnamese adolescents | ST & BT | Exclude - not qualitative |
| Tillman et al., 2018 | Understanding the experiences of middle school girls who have received help for non-suicidal self-injury | DR & BT | Include |
| Tingey et al., 2014 | Risk pathways for suicide among Native American adolescents | ST & DR | Include |
| Tirolla et al., 2020 | Clinical and epidemiological analysis of suicide attempts in children assisted by a poison control center | ST & DR | Exclude - not qualitative |
| Townsend et al., 2016 | Uncovering key patterns in self-harm in adolescents: Sequence analysis using the Card Sort Task for Self-harm (CaTS) | ST & GH | Exclude - not qualitative |
| Townsend et al., 2022 | Life problems in children and adolescents who self-harm: findings from the multicentre study of self-harm in England | ST & GH | Exclude - not qualitative |
| Vega et al., 2017 | Exploring the relationship between non suicidal self-injury and borderline personality traits in young adults | ST & BT | Exclude - not qualitative |
| Vergara et al., 2023 | Self-injury functions, romantic relationship stress, and suicide attempts in adolescents | ST & GH | Exclude - not qualitative |
| Verroken et al., 2018 | Starting from scratch: Prevalence, methods, and functions of non-suicidal self-injury among refugee minors in Belgium | ST & AS | Exclude - not qualitative |
| Victor et al., 2015 | Characteristics of nonsuicidal self-injury associated with suicidal ideation: Evidence from a clinical sample of youth | ST & BT | Exclude - not qualitative |
| Victor et al., 2018 | Understanding the social context of adolescent nonsuicidal self-injury | ST & BT | Exclude - not qualitative |
| WachterMorris et al., 2020 | Functions and prevalence of self-directed violence in adolescence | ST & DR | Exclude - not qualitative |
| Wadman et al., 2017 | An interpretative phenomenological analysis of the experience of self-harm repetition and recovery in young adults | DR & BT | Include |
| Wadman et al., 2017 | A sequence analysis of patterns in self-harm in young people with and without experience of being looked after in care | ST & AS | Exclude - not qualitative |
| Wadman et al., 2018 | An interpretative phenomenological analysis of young people's self-harm in the context of interpersonal stressors and supports: Parents, peers, and clinical services | ST & DR | Include |
| Wang et al., 2022 | The psychological characteristics and risk factors of suicidal attempt among mood disorders adolescents accompany with non-suicidal self-injury: A multi-center study | ST & BT | Exclude - not qualitative |
| Wang et al., 2024 | Can digital self-harm relate to suicidal thoughts and behaviors beyond physical self-harm? | AS & BT | Exclude - not qualitative |
| Westers et al., 2014 | An exploration of adolescent nonsuicidal self-injury and religious coping | ST & BT | Exclude - not qualitative |
| Williams et al., 2021 | Understanding the processes underlying self-harm ideation and behaviors within LGBTQ+young people: A qualitative study | ST & DR | Include |
| Wong et al., 2022 | The subjective experience of non-suicidal self-injury among female Chinese university students | ST & DR | Include |
| You et al., 2013 | Functions of nonsuicidal self-injury among Chinese community adolescents | ST & BT | Exclude - not qualitative |
| You et al., 2015 | Comparing among the experiences of self-cutting, hitting, and scratching in Chinese adolescents attending secondary schools: An interview study | ST & AS | Exclude - not qualitative |
| Zetterqvist et al., 2013 | Prevalence and function of non-suicidal self-injury (NSSI) in a community sample of adolescents, using suggested DSM-5 criteria for a potential NSSI disorder | ST & GH | Exclude - not qualitative |
| Zetterqvist et al., 2014 | A cross-sectional study of adolescent non-suicidal self-injury: Support for a specific distress-function relationship | ST & AH | Exclude - not qualitative |
| Zhang et al., 2019 | Non-suicidal self-injury in Shanghai inner bound middle school students | ST & BT | Exclude - not qualitative |
| Zhao et al., 2024 | Self-injury functions mediate the association between anxiety and self-injury frequency among depressed Chinese adolescents: sex differences | ST & WYZ | Exclude - not qualitative |
| Zhu et al., 2024 | Experiences and cognitive characteristics of non-suicidal self-injury in adolescents with depression: a qualitative study | ST & DR | Include |
| Zygo et al., 2019 | Prevalence and selected risk factors of suicidal ideation, suicidal tendencies and suicide attempts in young people aged 13-19 years | ST & BT | Exclude - not qualitative |
